# Identification of SARS-CoV-2 Spike Palmitoylation Inhibitors That Results in Release of Attenuated Virus with Reduced Infectivity

**DOI:** 10.3390/v14030531

**Published:** 2022-03-04

**Authors:** Ahmed A. Ramadan, Karthick Mayilsamy, Andrew R. McGill, Anandita Ghosh, Marc A. Giulianotti, Haley M. Donow, Shyam S. Mohapatra, Subhra Mohapatra, Bala Chandran, Robert J. Deschenes, Arunava Roy

**Affiliations:** 1 Department of Molecular Medicine, University of South Florida, Tampa, FL 33612, USA; ramadan@usf.edu (A.A.R.); karthick1@usf.edu (K.M.); armcgill@usf.edu (A.R.M.); anandita1@usf.edu (A.G.); smohapat@usf.edu (S.S.M.); smohapa2@usf.edu (S.M.); chandran@usf.edu (B.C.); 2 Department of Veterans Affairs, James A Haley Veterans Hospital, Tampa, FL 33612, USA; 3 Department of Internal Medicine, University of South Florida, Tampa, FL 33612, USA; 4 Center for Translational Science, Florida International University, Port St. Lucie, FL 34987, USA; mgiulian@fiu.edu (M.A.G.); hdonow@fiu.edu (H.M.D.)

**Keywords:** SARS-CoV-2, palmitoylation, S-acylation, spike, post-translational modifications, DHHC9, palmitoyltransferase, palmitoyltransferase inhibitor, bis-piperazine, antiviral

## Abstract

The spike proteins of enveloped viruses are transmembrane glycoproteins that typically undergo post-translational attachment of palmitate on cysteine residues on the cytoplasmic facing tail of the protein. The role of spike protein palmitoylation in virus biogenesis and infectivity is being actively studied as a potential target of novel antivirals. Here, we report that palmitoylation of the first five cysteine residues of the C-terminal cysteine-rich domain of the SARS-CoV-2 S protein are indispensable for infection, and palmitoylation-deficient spike mutants are defective in membrane fusion. The DHHC9 palmitoyltransferase interacts with and palmitoylates the spike protein in the ER and Golgi and knockdown of DHHC9 results in reduced fusion and infection of SARS-CoV-2. Two bis-piperazine backbone-based DHHC9 inhibitors inhibit SARS-CoV-2 S protein palmitoylation and the resulting progeny virion particles released are defective in fusion and infection. This establishes these palmitoyltransferase inhibitors as potential new intervention strategies against SARS-CoV-2.

## 1. Introduction

The last two decades have seen the emergence of three significant coronavirus (CoV) outbreaks, including the Severe Acute Respiratory Syndrome-CoV (SARS-CoV) in 2002, the Middle East Respiratory Syndrome-CoV (MERS-CoV) in 2012, and most recently, the SARS-CoV-2 in 2020. β-Coronaviruses are enveloped, positive-stranded RNA viruses that express a spike protein on their surface. The SARS-CoV-2 spike glycoprotein (S) is a 1273 amino acid, type I membrane protein that binds to the ACE2 receptor on the host cell surface to initiate infection, virus uptake, and cell–cell fusion [1,2]. The unprocessed S protein precursor consists of an N-terminal signal sequence for endoplasmic reticulum (ER) insertion and a large ectodomain (ER luminal-virion exterior) composed of glycosylation sites, a receptor binding domain (RBD), a trimerization domain, and two proteolytic cleavage sites (S1/S2 and S2′) that are required for the conformational changes that present a fusion peptide domain for membrane insertion [3,4]. On the cytosolic side of the single membrane spanning domain is a short endodomain that contains a cysteine-rich domain (CRD) that undergoes S-acylation/palmitoylation, the functional significance of which is under active investigation [5,6,7,8,9].

Protein palmitoylation is the reversible post-translational addition of palmitate, from palmitoyl-CoA, onto the side chain of cysteine residues via a thioester linkage [10]. The reaction is catalyzed by a family of palmitoyl-acyltransferases (PATs) containing a consensus DHHC (Asp-His-His-Cys) amino acid sequence [11,12]. While originally described as a fatty acid modification of the Sindbis virus and vesicular stomatitis virus (VSV) glycoproteins [13,14], and as a lipid anchor for peripheral membrane yeast proteins Ras2 and Yck2 [15,16], palmitoylation is now known to be a common post-translational modification that occurs on more than 30% of all cellular proteins [17]. Owing to its reversible nature, palmitoylation plays major regulatory roles in protein membrane association and distribution, subcellular trafficking, vesical fission and fusion, and protein stability [18,19]. Palmitoylation of viral proteins has been implicated in viral genome replication, virion assembly, budding, and cell fusion [20]. For example, palmitoylation of murine coronavirus (MHV) spike is essential for virion assembly and infectivity [21], and palmitoylation-deficient recombinant MHV viruses are deficient in cell fusion and syncytia formation [22]. Similar results have also been observed in MERS and SARS-CoV [23,24]. In Coronaviruses, palmitoylation of the spike protein occurs during its maturation through the secretory pathway, namely in the ER and the Golgi [24], resulting in its localization in cholesterol and sphingolipid-rich membrane domains where viral budding is initiated [8,25]. In addition, a fraction of the spike protein traverses the secretory pathway to the plasma membrane independent of virion particles where it can bind ACE2 on uninfected cells leading to formation of multinucleated syncytia [26,27]. This contributes to the cell–cell spreading of the virus without the release of virion particles [28].

Targeting the protein, palmitoylation machinery has been suggested as a potential antiviral strategy and this is supported by recent metabolic modelling studies [29,30]. However, efforts to identify specific DHHC enzyme–substrate pairs are in the early stages. Several groups have attempted to identify the DHHC palmitoyltransferase or palmitoyltransferases for the SARS-CoV-2 S protein [5,6,8,31]. There appear to be several candidates. For example, Wu et al. and others have indicated that DHHC5 is associated with the palmitoylation of the SARS-CoV-2 S protein [6,32]. In addition, pull-down experiments by Gordon et al. using the SARS-CoV-2 S protein as bait detected DHHC5 palmitoyltransferase and its auxiliary protein, Golga7 [31]. In contrast, Mesquita et al. recently reported that the SARS-CoV-2 S protein is sequentially palmitoylated by DHHC20, DHHC9 and 8 [8], whereas Puthenveetil et al. identified DHHCs 2, 3, 6, 11, 20, 21, and 24 as putative S-acylation enzymes for the SARS-CoV-2 S protein [5]. These reports invoke the notion that in-depth knowledge of the S protein palmitoylation inhibition mechanisms could serve as the foundation for an efficient antiviral drug development program [29]. However, the most widely used inhibitor, 2-bromopalmitic acid (2-BP), is a non-specific and non-metabolizable palmitate analog [33] that also inhibits other unrelated lipid metabolizing enzymes [34]. Previously, we demonstrated two novel PAT inhibitors based on a bis-piperazine scaffold that had a *K_i_* one tenth that of 2-BP [35], suggesting that these bis-piperazine derivatives are at least an order of magnitude more specific than 2-BP. In this report, we show that these novel compounds inhibit palmitoylation of the SARS-CoV-2 spike protein, resulting in reduced virus infectivity.

The present study investigates the role of palmitoylation on the SARS-CoV-2 S protein beginning with a pseudotyped luciferase lentivirus system, followed by studies with infectious SARS-CoV-2 viruses. We created a series of S protein cysteine mutants and demonstrate that the S protein is palmitoylated on multiple cysteine residues, and mutation of these cysteine clusters adversely affects pseudovirus entry and cell–cell syncytium formation. We then evaluate the role of palmitoylation in the membrane localization, ACE2 receptor binding, cell–cell syncytia formation, and infection of the S protein. Next, we show that the DHHC9 palmitoyltransferase plays a major role in palmitoylating S protein and inhibition of DHHC9, inhibits S palmitoylation, reduces SARS-CoV-2 infectivity and inhibits cell–cell syncytia formation. Finally, we demonstrate that two novel bis-piperazine backbone-based protein S-palmitoylation inhibitors efficiently and selectively inhibit SARS-CoV-2 S protein palmitoylation, infection, and syncytia formation. Together, these results support the idea that the cellular protein palmitoylation machinery is a potential “druggable” target for SARS-CoV-2 infections.

## 2. Materials and Methods

### 2.1. Plasmids and HIV-1-Derived Pseudovirus Generation

Plasmids used in this study for the production of pseudotyped virus were a gift from Jesse D. Bloom’s lab (BEI Resources, Manassas, VA, USA, #NR52516, NR52517, NR52518 and NR52519). The HDM-IDTSpike-fixK (BEI catalog number NR-52514) plasmid expressing a codon-optimized S protein from SARS-CoV-2 strain Wuhan-Hu-1 (Genbank, Bethesda, MD, USANC_045512) under a CMV promoter, was used as the wild-type S protein and we performed all the mutagenesis on this codon-optimized S protein clone. Third generation lentivirus EGFP plasmid, pLJM1-EGFP, was a gift from David Sabatini (Addgene, Watertown, MA, USA, #19319). Third generation lentivirus was produced using the flowing packaging plasmids, which were a gift from Didier Trono’s lab, pMDL (Addgene, Watertown, MA, USA, #12251), pRev (Addgene, Watertown, MA, USA, #12253) and pMD2.G (Addgene, Watertown, MA, USA, #12259) [36].

HIV-1-derived virus particles pseudotyped with full length wild-type and mutant SARS-CoV-2 S protein were generated by transfecting HEK293T cells as previously described [37]. Briefly, plasmids expressing the HIV-1 gag and pol (pHDM-Hgpm2), HIV-1 rev (pRC-CMV-rev1b), HIV-1 Tat (pHDM-tat1b), the SARS-CoV-2 S protein (pHDM-SARS-CoV-2 Spike) and a luciferase/ZsGreen reporter (pHAGE-CMV-Luc2-IRES-ZsGreen-W) were co-transfected into HEK293T cells at a 1:1:1:1.6:4.6 ratio using CalPhos mammalian transfection kit (TaKaRa Clontech, Mountain View, CA, USA, #631312) according to manufacturer’s instructions. Fresh media were added after 18 h and 60 h later, culture supernatant was collected, clarified by passing through 0.45 um filter and used fresh.

### 2.2. Cell Culture

Human female embryonic kidney HEK293T cells (ATCC) were grown in Dulbecco modified Eagle’s medium (DMEM) supplemented with 10% fetal bovine serum (FBS) (Sigma, St. Louis, MO, USA, #F4135) and Penicillin-Streptomycin (Gibco, Billings, MT, USA, #15140148). HEK293T cells constitutively expressing human ACE2 (HEK293T-ACE2 cells) obtained from BEI Resources, Manassas, VA, USA. (#NR52511) were grown in the same media supplemented with hygromycin (100 μg/mL). HEK293T cells constitutively expressing EGFP (HEK293T-EGFP) were established by transducing HEK293T cells with the pLJM1-EGFP containing lentivirus. Lentivirus was produced by transfecting 10^6^ HEK293T cells on a 60 mm plate with pMDL, pRev and pVSVG using CalPhos mammalian transfection kit (TaKaRa Clontech, Mountain View, CA, USA). Next day, fresh media were added, then two days after transfection, the supernatant was harvested, and filtered with a 0.45 μm filter to remove cell debris. The filtered supernatant containing lentiviral vectors was used to transduce HEK293T cells seeded one day before. Then, 48 h later, the cells were selected in DMEM supplemented with 2 μg/mL puromycin for one week. The puromycin-resistant population of HEK293T-EGFP cells was found to constitutively express EGFP by fluorescent microscopy. These HEK293T-EGFP cells were used as donor cells in syncytium formation assays. Caco-2 (human colon epithelial cells) cells, obtained from ATCC (HTB-37), were grown in Minimum Essential Medium (Gibco, Billings, MT, USA, #11095080). Media were supplemented with 20% fetal bovine serum (Sigma, St. Louis, MO, USA, #F4135), Penicillin-Streptomycin (Gibco, Billings, MT, USA, #15140148), 1× non-essential amino acid solution (Cytiva, Marlborough, MA, USA, #SH3023801) and 10 mM sodium pyruvate (Gibco, Billings, MT, USA, #11360070). All cell lines were incubated at 37 °C in the presence of 5% CO_2_.

### 2.3. CoV S Protein Sequence Alignment

Amino acid sequences of the S protein used in the alignment were obtained from UniProKB. The accession numbers are SARS-CoV-2 (P0DTC2), SARS-CoV-1 (P59594), Bat RaTG13 (A0A6B9WHD3), MERS (K9N5Q8), MHV-A59 (P11224), HCoV-OC43 (P36334), HCoV-229E (P15423), TGEV (P07946) and IBV (P11223). Alignment of these sequences was performed using Clustal Omega (https://www.ebi.ac.uk/Tools/msa/clustalo/ (accessed on 10 May 2020)).

### 2.4. Site-Directed Mutagenesis

All mutagenesis was carried out using Q5 Site-directed Mutagenesis Kit Protocol (NEB, #E0554S) according to the manufacturer’s instructions using primers in Table 1. All mutagenesis was confirmed with sequencing (GENEWIZ, South Plainfield, NJ, USA).

### 2.5. Transfection

For pseudotyped virus production, HEK293T cells were transfected using the CalPhos mammalian transfection kit (TaKaRa Clontech, Mountain View, CA, USA) according to the manufacturer’s instructions. For the syncytia formation assay, HEK293T-GFP cells were transiently transfected with wild-type and mutant S plasmids using TransIT-X2 transfection reagent (Mirus, Madison, WI, USA, #MIR 6000) according to the manufacturer’s instructions, and fresh media were added after 6 h.

### 2.6. SARS-CoV-2 S Protein Pseudotyped Virus Entry

The assay was conducted as previously described [37] with minor modification. Briefly, 96 flat bottom well plates were coated with poly-D-lysine (Gibco, Billings, MT, USA, #A3890401) and seeded with 1.25 × 104 HEK293T-ACE2 or Caco-2 cells per each well. After 12 h, pseudotyped virus with wild-type and mutant S protein (with no polybrene) were used for infection. After 48 h, Steady-Glo Reagent (Promega, Madison, WI, USA, #E2620) equal to the volume of culture medium in each well was added as per manufacturer’s instructions, cells were allowed to lyse for 5 min and then luminescence measured with a microtiter plate reader (Biotek, Winooski, VT, USA).

### 2.7. Pseudovirus Egress

In order to quantitate HIV-based pseudovirus egress from HEK293T cells, we performed ELISA against HIV p24 (TaKaRa Clontech, Mountain View, CA, USA, #632200) according to manufacturer’s instructions. Briefly, pseudotyped virus containing cell culture supernatant was collected, diluted 1:20, ELISA performed, and absorbance measured at 450 nm using a microtiter plate reader (BioTek, Winooski, VT, USA).

### 2.8. Immunoblotting

Whole cell lysates were prepared using RIPA Lysis Buffer (Thermo Scientific, Waltham, MA, USA, #89900) supplemented with a protease inhibitor cocktail (Roche, Mannheim, Baden-Württemberg, Germany# 11836170001) for 30 min on ice and then sonicated three times at an amplitude setting of 50 with pulses of 15 s on and 15 s off on a Qsonica Q700 sonicator (Newtown, CT, USA). The lysates were clarified by centrifugation at 13,000× *g* for 15 min at 4 °C. Protein concentrations were estimated using Pierce BCA protein assay kit (Thermo Scientific, Waltham, MA, USA, #23225) as per manufactural instructions and equal concentration of proteins resolved appropriate SDS PAGE gels. SDS polyacrylamide gels were transferred on Nitrocelluose membranes (GE) by Wet transfer Bio-Rad System at 300 Amps for 90 min at 4 °C, membranes were blocked with 5% non-fat milk for 1 h at room temperature (RT) and were incubated with primary antibodies diluted in PBST solution at 4 °C overnight. All primary antibodies used in this study are listed in Table 2. Membranes were washed with PBST for 5 min, 3 times and subsequently incubated with appropriate secondary antibodies. Blots were then washed three times with PBST for 5 min each wash. The immunoreactive bands were developed using Super Signal West Pico chemiluminescent substrate (Thermo Scientific, Waltham, MA, USA, #34078) or Super Signal West Femto chemiluminescent substrate (Thermo Scientific, Waltham, MA, USA, #34095) depending on the signal strength. Blots were developed on a Bio-Rad ChemiDoc XRS + System.

### 2.9. Co-Immunoprecipitation (Co-IP)

Whole cell lysates were prepared using Pierce IP Lysis Buffer (Pierce, Waltham, MA, USA, #87788) supplemented with a protease inhibitor cocktail for 30 min on ice and then sonicated three times at an amplitude setting of 30 with pulses of 15 s on and 15 s off on a Qsonica Q700 sonicator at 4 °C. The lysates were then clarified by centrifugation at 13,000× *g* for 15 min at 4 °C and protein concentrations estimated by BCA reaction. For IP, 300 µg of the prepared lysate was incubated with the appropriate antibody (3 µg) and pulled down using protein A Sepharose 6MB (GE healthcare, Chicago, IL, USA, #17-0469-01). All primary antibodies used in this study are listed in Table 2. The immunoprecipitates were washed three times with lysis buffer at 4 °C and resolved on SDS-PAGE followed by immunoblotting. Wherever mentioned, light-chain-specific secondary antibodies were used to avoid heavy chain bands in WB of Co-IP experiments.

### 2.10. Acyl-PEGyl Exchange Gel-Shift (APEGS) Assay

To assess the level of protein S-palmitoylation on the SARS-CoV-2 S protein we conducted APEGS as described previously [38]. In brief, HEK293T cells transfected with appropriate plasmids were lysed with the following buffer, 4% SDS, 5 mM EDTA, in triethanolamine buffer (TEA) pH 7.3 with protease inhibitors and PMSF (5 mM). After centrifugation at 20,000× *g* for 15 min, proteins in the supernatant were reduced with 25 mM tris(2-carboxyethyl)phosphine (TCEP, Thermo Scientific, Waltham, MA, USA, #20490) for 1 h at RT, and free cysteine residues were blocked with 20 mM N-ethyl maleimide (NEM, Sigma, St. Louis, MO, USA, #E3876) for 3 h at RT. To terminate the NEM reaction and wash any residual NEM, pre-chilled methanol: chloroform: H_2_O (4:1.5:3) was added to the reaction. This wash was repeated three times. Next, the proteins were re-suspended in TEA with 4% SDS and 5 mM EDTA, then incubated in buffer containing 0.2% Triton X-100, 5 mM EDTA, 1 M NH_2_OH, pH 7.0 for 1 h at RT to cleave palmitoylation thioester bonds. The reaction was terminated as above, and proteins re-suspended in TEA with 4% SDS were PEGylated with 1.33 mM mPEGs (10 kDa, Sunbright, White Plains, NY, USA, #ME050) for 2 h at RT to label palmitoylation sites. The reaction was terminated as above; proteins were re-suspended with TEA buffer with 4% SDS. Protein concentration was measured by BCA protein assay. Thereafter, SDS PAGE sample buffer was added, and samples were heated at 70 °C for 10 min and run on a 7.5% gel for Spike and 12% for GAPDH.

### 2.11. Syncytium Formation Assay

HEK293T-EGFP cells (donor cell) were transiently transfected with wild-type and mutant S plasmids using TransIT-X2 transfection reagent (Mirus, Madison, WI, USA, #MIR 6000). HEK293T-ACE2 were stained with CellTracker Red CMTPX Dye (Invitrogen, Waltham, MA, USA, #C34552) according to manufacturer’s instructions. Then, 6 h after transfection, the HEK293T-EGFP cells were detached using Accutase (Sigma, St. Louis, MO, USA, #A6964) and overlaid on the CellTracker Red stained HEK293T-ACE2 cells, at a 1:1 ratio. Cells were fixed and imaged 48 h after transfection using Keyence BX700 fluorescent microscope. Average areas of the syncytia were quantified using ImageJ.

### 2.12. Compound Synthesis and Characterization

Bis-piperidines **13** and **25** were synthesized using solid-phase chemistry, purified by RP-HPLC, and characterized by LCMS and 1H NMR as previously described [35]. The compounds were additionally recharacterized by LCMS before performing all biological assays. LCMS analysis utilized a Shimadzu 2010 LCMS system, consisting of an components sourced from Kyoto, JP including a DGU-20A degasser unit and an SIL-20A HT autosampler, as well as components sourced from Canby, OR, USA including an LC-20AD binary solvent pump, and a CTO-20A column oven. A Shimadzu SPD-M20A diode array detector, sourced from Canby, OR, USA, was used for detections. A full spectra range of 190–800 nm was obtained during analysis. Chromatographic separations were obtained using a Phenomenex Gemini NX-C18 analytical column (5 μm, 50 × 4.6 mm ID). The column was protected by a Phenomenex Gemini-NX C18 column SecurityGuard (5 μm, 4 × 3.0 mm ID). All equipment was controlled and integrated by Shimadzu LCMS solutions software v 3. Mobile phases for LCMS analysis were HPLC grade or LCMS grade obtained from Fisher Scientific (Fair Lawn, NJ, USA). The mobile phases consisted of a mixture of LCMS grade acetonitrile and water (both with 0.1% formic acid for a pH of 2.7). The initial setting for analysis was at 5% acetonitrile (*v*/*v*), linearly increasing to 95% acetonitrile over 6 min. The gradient was then held at 95% acetonitrile for 2 min until linearly decreasing to 5% over 1 min. From there, the gradient was held until stop for an additional 3 min. The total run time was equal to 12 min. The total flow rate was set to 0.5 mL/minute. The column oven and flow cell temperature for the diode array detector were set at 40 °C. The auto sampler temperature was held at 15 °C, and 10 μL was injected for analysis.

4-(((2S)-1-(2-(4-isobutylphenyl) propyl)-4-(4-((2S)-1-(2-(4-isobutylphenyl) propyl) piperazin-2-yl) butyl) piperazin-2-yl) methyl) phenol (Compound **13**) OC1=CC=C(C[C@@H]2N(CC(C3=CC=C(CC(C)C)C=C3)C)CCN(CCCC[C@@H]4N(CC(C5=CC=C(CC(C)C)C=C5)C)CCNC4)C2)C=C1 LCMS (ESI+). Calculated exact mass for C45H68N4O: 680.54 found [M+H]^+^:681.55. Retention Time: 4.78 min. 90.1% purity by 214 nM.

4-(((2S)-4-(4-((S)-1-(3,5- bis(trifluoromethyl)phenethyl) piperazin-2-yl) butyl)-1-(2-(4-isobutylphenyl) propyl) piperazin-2-yl) methyl) phenol (Compound **25**) OC1=CC=C(C[C@@H]2N(CC(C3=CC=C(CC(C)C)C=C3)C)CCN(CCCC[C@@H]4N(CCC5=CC(C(F)(F)F)=CC(C(F)(F)F)=C5)CCNC4)C2)C=C1 LCMS (ESI+). Calculated exact mass for C42H56F6N4O: 746.44, found [M+H]^+^:747.40. Retention Time: 4.722 min. 90.4% purity by 214 nM.

### 2.13. Cellular Toxicity Assay

The XTT Cell Proliferation Kit II (Roche, Mannheim, Baden-Württemberg, Germany, #11465015001) was used for assessing cellular toxicity following manufacturer’s instructions. DMSO was used as a vehicle for all compounds and appropriate dilution of DMSO only was used as a control.

### 2.14. Gene Silencing

Genes were silenced using siRNAs obtained from IDT, DHHC5 (IDTDNA, Coralville, IA, USA, #290128941) and DHHC9 (IDTDNA, Coralville, IA, USA, #290128950). For each gene, we used a combination of three siRNAs. HEK293T cells were seeded on 24-well plate 24 h before transfection. Transfection of these siRNAs was performed using TransIT-X2 (Mirus, Madison, WI, USA, #MIR 6000) according to the manufacturer’s instructions. Silencing efficacy was checked using qPCR using SYBR Green real-time reagents (Invitrogen, Waltham, MA, USA, # 4367659) and immunoblotting.

### 2.15. RNA Extraction and RT-qPCR

Total RNA was isolated using the RNeasy minikit (Qiagen, Hilden, Germany, #74106) following manufacturer’s instructions. On-column DNase digestion was performed by using an RNase-free DNase set (Qiagen, Hilden, Germany, #79254). The extracted RNA concentration was estimated using a NanoDrop spectrophotometer (Thermo Scientific, Waltham, MA, USA), and 1 μg RNA was reverse transcribed by using the High-Capacity cDNA reverse transcription kit (Applied Biosystems, Waltham, MA, USA, #4368814) with random primers, according to the manufacturer’s instructions. For real-time quantitative reverse transcription-PCR (qRT-PCR), the synthesized cDNA was diluted 1:20 and used as a template with Power SYBR Green PCR Master Mix (Applied Biosystems, Waltham, MA, USA, #4367659) on an ABI Prism 7500 detection system (Applied Biosystems, Waltham, MA, USA). All RNA levels were normalized to β-actin mRNA levels and calculated as the delta-delta threshold cycle (ΔΔCT). Primers used in this study are listed in Table 3.

### 2.16. Immunofluorescence Assay (IFA) and Proximity Ligation Assay (PLA)

Cells were grown on eight-chamber glass slides and treated as described in the results. After appropriate incubations, the cells were fixed using 4% paraformaldehyde for 15 min, and permeabilized with 0.2% Triton X-100 in PBS for 20 min. The slides were then washed, blocked with Image-iT FX signal enhancer (Invitrogen, Waltham, MA, USA, #I36933) for 30 min at 37 °C and incubated with primary antibodies for 1 h at 37 °C. All primary antibodies used in this study are listed in Table 2. After this, the slides were washed three times in PBS and incubated with corresponding fluorescent dye-conjugated secondary antibodies for 30 min at 37 °C. PLA was performed according to the manufacturer’s instructions using the following kits and reagents: Duolink in situ PLA Probe Anti-Rabbit PLUS (Sigma-Aldrich, St. Louis, MO, USA, #DUO92002), Duolink in situ PLA Probe Anti-Mouse MINUS (Sigma-Aldrich, St. Louis, MO, USA, #DUO92004), Duolink in situ Detection Reagents Red (Sigma-Aldrich, St. Louis, MO, USA, #DUO92008). After completion of IFA or PLA, slides were mounted using mounting medium containing DAPI and observed either by a Keyence BZ-X fluorescence microscope.

### 2.17. Surface Immunofluorescence Assay

To detect the amount of S protein localizing to the surface of the cell, HEK293T cells were seeded in 8-chamber glass bottomed slides and transfected with appropriate plasmids. Cells were washed and treated with freshly prepared 0.1% paraformaldehyde and incubated for 10 min at 4 °C. Thereafter, the cells were washed two times with DMEM with 5% FBS and incubated with anti-S antibody diluted (1:200) in DMEM with 5% FBS and 0.1% Sodium Azide for 1 h at 37 °C. Next, cells were washed four times with DMEM with 5% FBS at 4 °C for 10 min and incubated with appropriate secondary antibodies (1:500) for 30 min at 4 °C. Finally, the cells were washed, anti-fade reagent added and imaged using a Keyence BX700 (Osaka, Japan) microscope.

### 2.18. Spike Surface Abundance by Flow Cytometry

The procedure was performed as described in [27] with minor modifications. Briefly, U2OS cells were seeded at a density of 8 × 104 cells/cm^2^ in 60 mm plates. Cells were transfected with 6 μg of plasmid DNA encoding different Spike WT and mutants, using TransIT-X2 (Mirus, Madison, WI, USA, #MIR6000). Cells were washed 24 h after infection once in PBS and dissociated from the flask using Accutase (Sigma, St. Louis, MO, USA, #A6964) for 8 min at 37 °C. Cells were then washed once in ice cold FACS buffer (2% FCS in PBS) by re-suspension and centrifugation at 300× *g* for 5 min at 4 °C. The supernatant was removed, and cells were re-suspended in FACS buffer containing an anti-Spike (1:500, GeneTex, Irvine, CA, USA, #901509) and an eFluor 780 fixable viability dye (Thermo Fischer Scientific, Waltham, MA, USA, #65-0865-14). Cells were incubated on ice, in darkness for 1 h. Next, cells were washed 3 times in FACS buffer and incubated in Alexa Fluor 594 anti-mouse for 30 min, on ice. Then cells were then washed 3 times with FACS buffer and incubated in Cyto-Fast Fix/Perm Buffer (BioLegend, San Diego, CA, USA, #426803) for 20 min at room temperature. Following this, cells were washed once in Cyto-Fast Wash Buffer (BioLegend, San Diego, CA, USA, #426803) and incubated in Cyto-Fast Wash Buffer containing anti-Spike Ab (1:500, GeneTex Irvine, CA, USA, #901509) for 30 min at room temperature. Cells were then washed 3 times with Cyto-Fast Wash Buffer and re-suspended in Cyto-Fast Wash Buffer containing Alex-fluor 488. Finally, cells were washed twice and re-suspended in FACS buffer, and analyzed using FLOW cytometry. The percentage of cells expressing surface S protein was normalized to the percentage of cells expressing total (intracellular + surface) S protein.

### 2.19. SARS-CoV-2 Virus Stock Preparation and Titration with Plaque-Based Assays

All replication-competent SARS-CoV-2 experiments were performed in a biosafety level 3 laboratory (BSL-3) at the University of South Florida. All viral stocks were produced and isolated from supernatants of Vero-E6 ACE2 cells, cultured in T175 culture flasks to a confluency of 80–90%, and infected with an original passage 2 (P2) SARS-CoV-2 or SARS-CoV-2-mNG (SARS-CoV-2 stably encoding mNeonGreen) virus, at MOI of 0.1 for 72 h, in 10 mL MEM supplemented with 5% FBS. SARS-CoV-2 was obtained from BEI Resources (Manassas, VA, USA, #NR52281), while SARS-CoV-2-mNG was a kind gift from Dr. PEI-Yong Shi from the University of Texas Medical Branch, Galveston, TX, USA [41]. Supernatants were harvested, cleared of cell debris by centrifugation (500× *g*, 10 min) and filtration (0.45 μm), mixed with 10% SPG buffer (ATCC, Manassas, VA, USA, #MD9692), aliquoted and stored at −80 °C. Viral titers were quantified by determining the number of individual plaque forming units after 72 h of infection on confluent Vero-E6-ACE2 expressing cells. In brief, viral stocks were serially diluted (10-fold) in serum-free medium and inoculated on 1× 105 Vero-E6-ACE2 cells in triplicates in a 48 well plate.

### 2.20. SARS-CoV-2 Infection

All SARS-CoV-2 infections were performed using the same passage 3 SARS-CoV-2 or SARS-CoV-2-mNG virus stocks. Caco-2 cells seeded to a confluency of 70 to 80% were washed twice in warm serum-free medium and inoculated with the indicated MOI of the appropriate virus, diluted in serum-free medium (5 mL for T75; 2 mL for T25; 1 mL for 6-well plates). Two hours after inoculation cells were washed with complete medium and infection was allowed to proceed for the indicated time points in DMEM supplemented with 2.5% FBS. After infection, media with respective drugs were added and incubated for 72 h. Images were quantified using ImageJ (v.1.53f51).

### 2.21. Drug Treatments

For drug treatment, cells were treated with indicated concentrations of 2-BP (Sigma, St. Louis, MO, USA, #238422) or compounds **13** and **25** dissolved in DMSO, 12 h prior to infection. Post-infection, cells were continued to be incubated in presence of the respective drugs for the indicated time.

### 2.22. Statistical Analysis and Reproducibility

Statistical analysis was performed using GraphPad Prism 9 (San Diego, CA, USA). For two groups, means were compared by a two-tailed unpaired Student’s *t*-test. For multiple groups, analysis was performed by One-Way ANOVA with Dunnett correction for multiple comparisons. *p* value of <0.05 was considered statistically significant. Specific statistical test results are indicated in each figure: * = *p* < 0.05; ** = *p* < 0.01; *** = *p* < 0.001; **** = *p* < 0.0001.

## 3. Results

### 3.1. SARS-CoV-2 S Protein Is Palmitoylated on Multiple Sites

**Figure 1:**
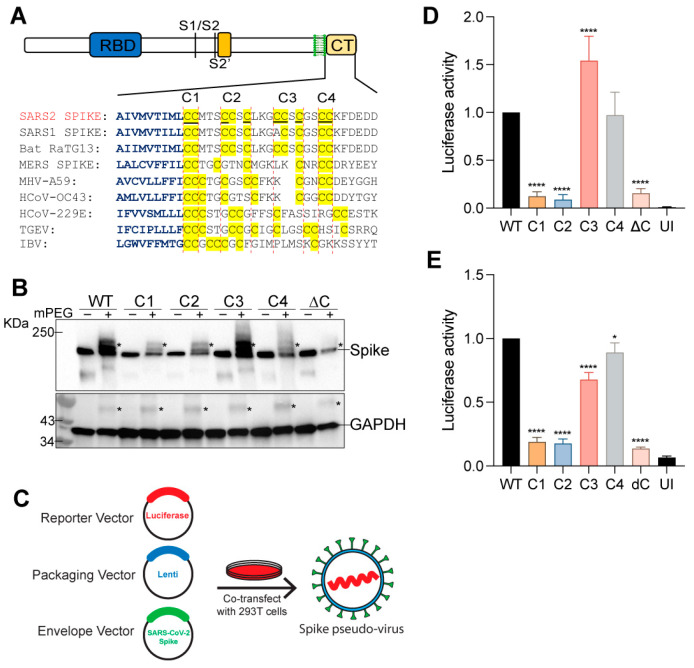
Palmitoylation of SARS-CoV-2 S protein and effect of different cysteine cluster mutations of the S protein on pseudotyped lentivirus infection. (**A**) Sequence alignment of the carboxy-terminal tails of the S protein from the indicated Coronaviruses. The transmembrane region residues are depicted in blue and the conserved cysteine residues in the C-terminal cysteine-rich domain (CRD) are highlighted in yellow. The 10 cysteines of the SARS-CoV-2 S protein (S) CRD were grouped as four clusters—C1 (C1235, 1236), C2 (C1240, C1241, 1243), C3 (C1247, 1248, 1250) and C4 (C1253, 1254). Palmitoylation site prediction algorithm predicted 9 of the 10 underlined cysteine residues as potential sites of palmitoylation (http://csspalm.biocuckoo.org/online.php (accessed on 10 August 2020)). (**B**) Plasmids with WT SARS-CoV-2 S protein (S) or the indicated cysteine mutants (C1-4 and ∆C, where all 10 cysteines are mutated to serine) were transfected into HEK293T cells and 48 h later, palmitoylation of the S protein was assessed by the Acyl-PEGyl Exchange Gel-Shift (APEGS) assay which employs mPEG-maleimide alkylation to label palmitoylated cysteine residues to study the effect of these mutations on the palmitoylation of the S protein. After PEGylation, the samples were separated by SDS–PAGE and visualized by chemiluminescence using a monoclonal antibody specific for the S2 fragment of the spike protein. The mPEG minus APEGS reactions served as a control and confirmed the observed gel-shifted palmitoylated bands were due to PEGylation at the available cysteine residues. Addition of mPEG results in slower migrating species, indicated by an asterisk (top panel). GAPDH serves as a loading and palmitoylation control (bottom panel). (**C**) Schematic of the luciferase reporter SARS-CoV-2 S protein pseudotyped lentivirus system used. (**D**) HEK293T-ACE2 or Caco-2 (**E**) cells were infected with lentivirus pseudotyped with WT S protein or its cysteine cluster mutants for 48 h and pseudovirus infection measured by quantifying the luciferase signal. UI represents uninfected control. Data shown are relative to the WT pseudovirus and are averages of the results of at least three independent experiments ± SD. (* *p* < 0.05; **** *p* < 0.0001 (One-Way ANOVA)).

Juxtamembrane cysteine residues of transmembrane proteins have a high propensity to be palmitoylated [42]. The cytoplasmic tail of the SARS-CoV-2 S protein has 10 highly conserved cysteine residues, 9 of which (underlined, Figure 1A) are predicted to be potential sites of palmitoylation by the palmitoylation prediction server at http://csspalm.biocuckoo.org/online.php (accessed on 10 August 2020) [43] (Figure 1A). To investigate the role of palmitoylation at these 10 cysteine residues, we grouped them into 4 clusters—C1 (C1235, 1236), C2 (C1240, C1241, 1243), C3 (C1247, 1248, 1250) and C4 (C1253, 1254), and mutated each cluster to serine (Figure 1A). We also generated a ∆C mutant in which all 10 cysteines were mutated to serine. The Acyl-PEGyl Exchange Gel-Shift (APEGS) assay was used to assess the palmitoylation of the S protein. We observed that the C1, C2, and C4 cysteine clusters reduced palmitoylation significantly, whereas the ∆C mutation led to undetectable levels of palmitoylation (Figure 1B). Mutating the C3 cluster appeared to be less important for palmitoylation (Figure 1B). The S protein shifted to several higher molecular weight bands representing different populations of the S protein with varying degrees of palmitoylation (Figure 1B). GAPDH, which is also known to undergo palmitoylation [44], was used as a positive control for the APEGS reaction and as a loading control to show equal protein loading per well. These observations are in agreement with recent reports [5,8].

### 3.2. Spike Protein Palmitoylation Is Required for Infection of Cells Expressing ACE2

Next, we employed a luciferase reporter SARS-CoV-2 S pseudotyped lentivirus system to study the effect of these cysteine cluster mutations on the cellular entry and infectivity of the S protein by measuring the luciferase activity 48 h after pseudovirus infection of HEK293T-ACE2 or Caco-2 cells (Figure 1C). Compared to WT S, the ability of the ∆C, C1, and C2 S mutant virus to infect HEK293T-ACE2 cells was significantly attenuated (Figure 1D). In contrast, clusters C3 and C4 mutations had little or no effect on pseudovirus infection. Similar results were observed with Caco-2 cells which express ACE-2 endogenously (Figure 1E). Together, these data indicate that palmitoylation of the first five cysteine residues of the SARS-CoV-2 S protein CRD (C1235, 1236, 1240, 1241, and 1243) are the most important residues for infection of ACE-2 expressing cells.

### 3.3. Effect of Palmitoylation on the Plasma Membrane Localization, Viral Egress, and ACE2 Binding of SARS-CoV-2 S Protein

**Figure 2:**
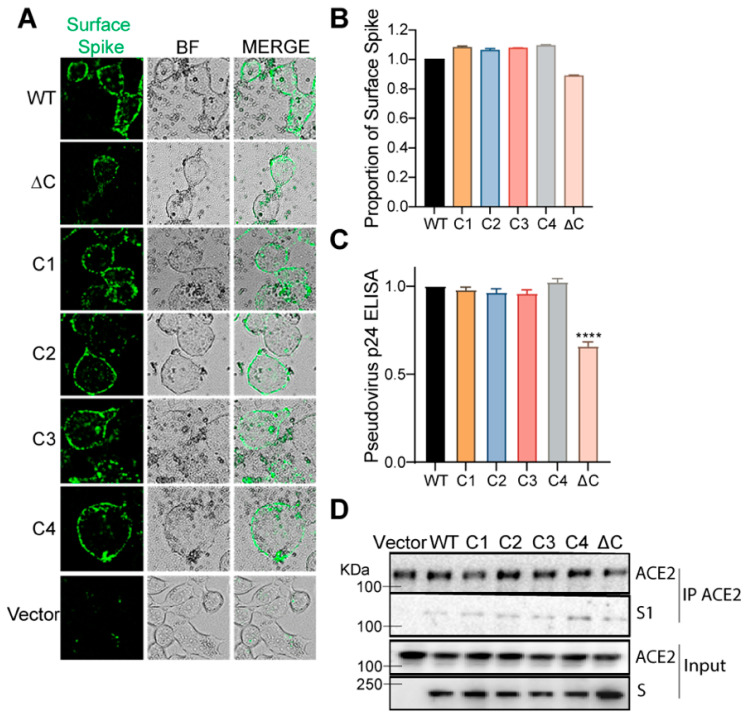
Effect of cysteine cluster mutations of SARS-CoV-2 S protein on its plasma membrane localization, egress and ACE2 binding. (**A**) Surface immunofluorescence assay for the S protein was performed by using anti-S protein antibodies on unpermeabilized HEK293T cells 48 h after transfection with WT or cysteine cluster mutant S proteins. BF indicates bright field. (**B**) FLOW cytometry assay measuring the ratio of surface expressed S protein vs. total intracellular S protein expressed. (**C**) HEK293T cells were transfected with plasmids for SARS-CoV-2 S protein pseudotyped lentivirus and 48 h later, supernatants containing pseudotyped lentivirus particles (WT and cysteine mutants) were assayed for pseudovirus egress by ELISA against the lentivirus (HIV) p24 protein. (**D**) Co-immunoprecipitation assay. Plasmids expressing WT or different cysteine cluster mutant S proteins were transfected into HEK293T-ACE2 cells. ACE2 was immunoprecipitated 48 h after transfection, and the presence of S protein was assayed by immunoblot using antibody specific to the S1 subunit. ACE2 immunoprecipitation was also confirmed. Inputs were quantitated by immunoblot (bottom panels). Data shown are averages of the results of at least three independent experiments ± SD. (**** *p* < 0.0001 (*t*-test)).

Lentivirus-based pseudotyped virus assembly and budding occurs at the host plasma membrane. To evaluate if the observed reduction in cellular entry and infectivity of the ∆C, C1, and C2 mutant S pseudotyped lentiviruses is due to defective membrane transport and localization, we investigated the effect of the cysteine cluster mutations on the transport of the S protein to the plasma membrane. For this, we performed surface immunofluorescence assay (SIF) and immunostaining of the S protein on non-permeabilized cells. We found that only the ∆C mutant exhibited a moderate reduction in its surface expression, while the rest of the cysteine cluster mutations had no measurable effect (Figure 2A). We confirmed these data with a FLOW cytometry-based assay, in which we immunostained surface S protein with Alexa Fluor 594 on non-permeabilized cells and then permeabilized the cells to stain the total intracellular S protein with Alexa Fluor 488. Dead cells were excluded from this assay using a fixable viability dye. The ration of surface vs. total S proteins was plotted, and the results confirmed that none of the cysteine mutants were defective in surface expression (Figure 2B and Figure S1).

Next, to ascertain that the observed reduction in the cellular entry and infectivity of the S mutants is not due to reduced pseudovirus egress and release, we measured the released WT and mutant S-pseudotyped virus. For this, we collected cell-free supernatants and measured S-pseudotyped lentivirus levels by ELISA against the lentivirus capsid protein, p24. Our results show that mutation of individual cysteine clusters had no measurable effect on the egress of the pseudotyped lentivirus (Figure 2C). However, mutating all of the cysteines (∆C) resulted in a moderate reduction, indicating that palmitoylation may contribute but is not required for pseudotyped lentivirus egress.

Next, the effect of S palmitoylation on ACE2 receptor binding was examined by co-immunoprecipitation. ACE2 was immunoreacted with anti-ACE2 antibody followed by detection of S by immunoblotting with anti-S antibody. There appeared to be no difference in ACE2 binding comparing WT to the cysteine cluster mutants (Figure 2D). Together, these experiments establish that palmitoylation of the S protein does not affect its intracellular trafficking, cell surface localization, pseudovirus egress, and ACE2 binding.

### 3.4. Palmitoylation Is Important for S Protein-Mediated Membrane Fusion and Syncytia Formation

**Figure 3:**
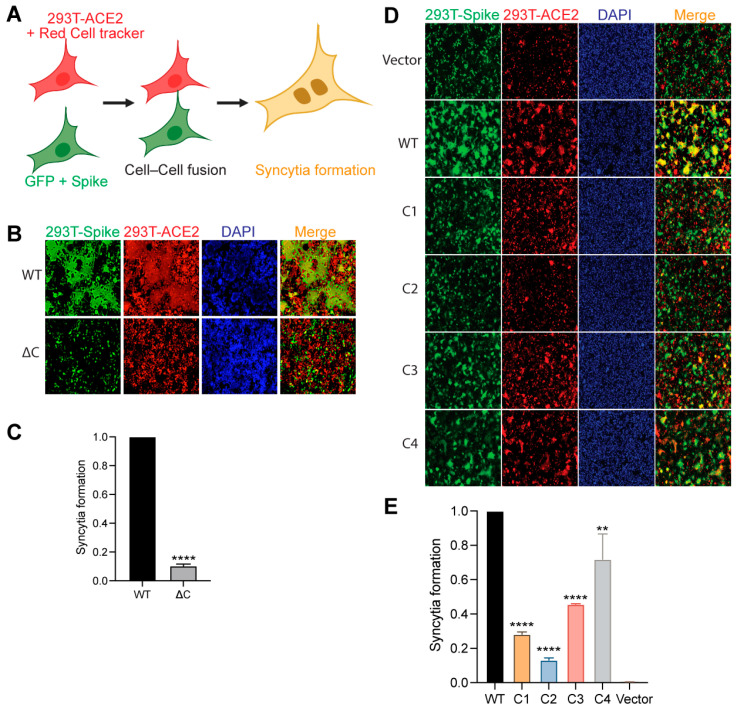
SARS-CoV-2 S protein palmitoylation is required for cell–cell fusion and syncytia formation. (**A**) Schematic representation of dual fluorescent syncytia formation assay. HEK293T-EGFP cells were transfected with WT S or its cysteine mutant plasmids. After 24 h, cells were detached and mixed with HEK293T-ACE2 cells labeled with cell tracker red CMPTX dye. After another 24 h, syncytia formation was evaluated by visualizing the yellow fluorescence formed by the fusion of the green and red cells. (**B**) Dual fluorescent syncytia formation assay with WT S and S∆C and (**C**) quantification of cell–cell fusion ability by measuring the pixel density of the observed syncytia. (**D**,**E**) Same as in B and C, but with cysteine cluster mutants of the S protein. Data shown are averages of the results of at least three independent experiments ± SD. (** *p* < 0.01; **** *p* < 0.0001 (*t*-test)).

CoV S proteins play a central role in mediating viral envelope fusion with a target cell membrane and fusion of infected and uninfected cells (syncytia formation) [45,46]. Both steps are essential for efficient virus entry and cell to cell spreading during pathogenesis. Therefore, we investigated the effect of the cysteine cluster mutations on the fusogenic potential of the SARS-CoV-2 S protein. For this, we transfected HEK293T-EGFP (HEK293T cells stably transfected with EGFP) with S WT or its mutant plasmids. Then, 293T-ACE2 cells were stained with a red cell tracker dye and 6 h later, the two cells were mixed at 1:1 ratio, and co-cultured together. When the GFP+S expressing cells and the ACE2 expressing red cells fuse and form a syncytium, it appears as yellow under a fluorescent microscope (Figure 3A). First, we compared S WT to the ΔC mutant and observed that the palmitoylation-deficient S protein exhibited a marked reduction in syncytia formation (Figure 3B,C). Subsequently, we tested all the cysteine cluster mutants, and consistent with our pseudovirus infection observations (Figure 1D,E), we observed that C1 and C2 cluster mutations have the greatest effect on syncytia formation (Figure 3D,E). C3 and C4 clusters had lower but significant effects on syncytia formation. These results collectively show that the first 5 cysteines of the SARS-CoV-2 S CRD (C1235, 1236, 1240, 1241, 1243) play the most important role in fusogenic activity.

### 3.5. DHHC9 PAT Is Instrumental in SARS-CoV-2 S Protein Palmitoylation

**Figure 4:**
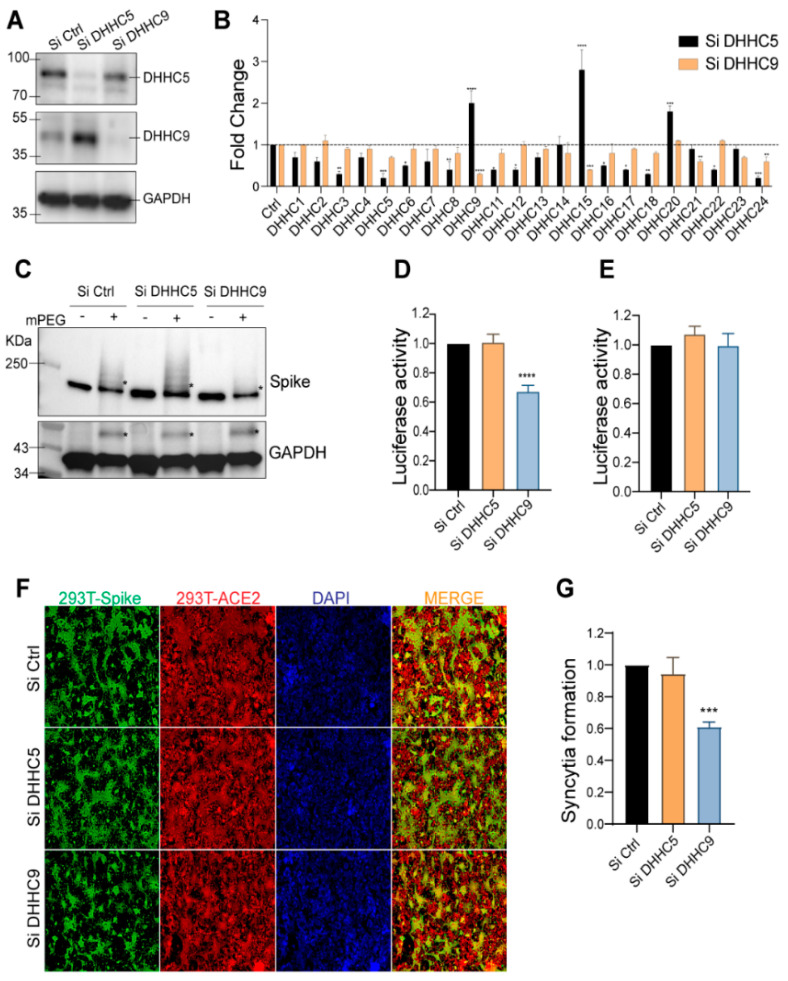
Evaluation of DHHC5 and DHHC9 acyltransferases as SARS-CoV-2 S protein palmitoylating enzymes. (**A**) DHHC5 and DHHC9 acyltransferases were knocked down using siRNA for 72 h in HEK293T cells and their respective protein levels were assessed by immunoblotting against the respective proteins. (**B**) qRT-PCR assessing the mRNA levels of 22 human PATs after DHHC5 and DHHC9 knocked down in HEK293T cells. (**C**) APEGS assay to evaluate the role of DHHC5 and DHHC9 on S protein palmitoylation in HEK293T cells. (**D**) HEK293T-ACE2 cells were infected with WT S protein pseudotyped lentivirus isolated from HEK293T cells treated with control siRNA or siRNA against DHHC5 or DHHC9. Results are normalized to control siRNA set at 1.0. (**E**) HEK293T-ACE2 cells were knocked down for DHHC5 or DHHC9 and 48 h later infected with WT S protein pseudotyped lentivirus derived from untreated HEK293T cells. Further 48 h later, pseudovirus infection was measured by quantifying the luciferase signal. Data shown are relative to the control siRNA. (**F**) Dual fluorescent syncytia formation assay. HEK293T-EGFP cells were transfected with siRNA targeting DHHC5 or DHHC9 and 48 h later, transfected WT-S plasmids. The cells were detached and mixed 24 h later with HEK293T-ACE2 cells labeled with cell tracker red CMPTX dye. After another 24 h, syncytia formation was evaluated by visualizing the yellow fluorescence formed by the fusion of the green and red cells. (**G**) Quantification of cell–cell fusion ability of the data described in (**E**) by measuring the pixel density of the observed syncytia. All data shown are averages of the results of at least three independent experiments (3 fields each) ± SD. * = *p* < 0.05; ** = *p* < 0.01; *** = *p* < 0.001, **** = *p* < 0.0001 (unpaired *t*-test).

With an aim to efficiently inhibit the S protein palmitoylation as a step towards developing a novel antiviral approach, we sought to identify a druggable DHHC PAT responsible for palmitoylating the S CRD. Recent pull-down experiments by Gordon et al. identified DHHC5 and Golga7 as interacting with the SARS-CoV-2 S protein [31]. Golga7 has been previously identified as an accessory protein of two DHHC enzymes, namely, DHHC9 and DHHC5 [47,48]. We thus knocked down DHHC5 and DHHC9 individually using siRNA in HEK293T cells, and 72 h later confirmed their knockdown efficiencies using WB (Figure 4A). We also measured mRNA levels of a panel of 22 DHHC genes following knockdown of DHHC5 and DHHC9 (Figure 4B). Interestingly, knockdown of DHHC5 led to a 1.8-fold upregulation of DHHC9, whereas knockdown of DHHC9 did not affect DHHC5 expression. Knockdown of DHHC5 also resulted in similar compensatory upregulation of DHHC15 and 20 (Figure 4B). However, knockdown of DHHC9 did not result in any such compensatory upregulation of any other PAT.

Subsequently, we used the APEGS assay to examine the palmitoylation of the S protein following knockdown of DHHC5 or DHHC9. Reduction of DHHC9 significantly reduced S palmitoylation (Figure 4C). In contrast, in DHHC5 knocked down cells, S protein palmitoylation increased presumably due to the increase in DHHC9, but we cannot rule out that DHHC15 and DHHC20 may also contribute since they are similarly upregulated in a DHHC5 knockdown (Figure 4B). As expected, palmitoylation of GAPDH was unaffected upon knockdown of either DHHC5 or DHHC9 [49] (Figure 4C, lower pane).

To evaluate the effect of DHHC5 and DHHC9 downregulation on the ability of S protein pseudotyped lentivirus to infect ACE2 expressing cells, we generated lentivirus particles from 293T cells in which DHHC5 and 9 were knocked down. Reduction of DHHC9, but not DHHC5, resulted in a significant reduction in infection of HEK293T-ACE2 cells (Figure 4D). In contrast, knockdown of DHHC9 or DHHC5 in the HEK293T-ACE2 cells had no effect on the infection by pseudovirus derived from untreated 293T cells (Figure 4E). Thus, S protein palmitoylation is required for infection of HEK293T-ACE2 recipient cells, but not for any downstream event following infection, such as endocytosis and release of viral RNA into the host cell. We next tested if syncytia formation requires DHHC5 or DHHC9. As seen in Figure 4F,G, knockdown of DHHC9, but not DHHC5, resulted in significantly reduced syncytial formation.

### 3.6. DHHC9 Co-Localizes and Interacts with the SARS-CoV-2 S Protein Both in Transfected and Infected Cells

**Figure 5:**
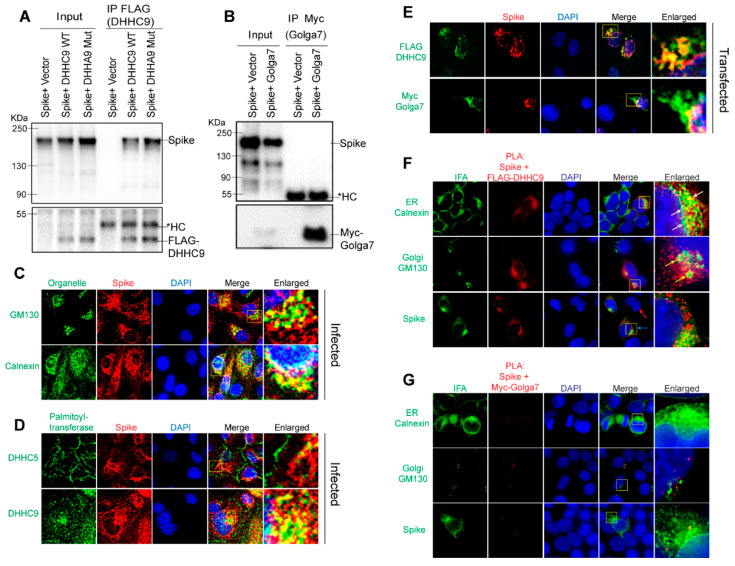
Interaction of DHHC9 with SARS-CoV-2 S protein. (**A**) Immunoprecipitation of FLAG tagged DHHC9 with S protein. HEK293T cells were transfected with either FLAG-DHHC9, or its catalytic inactive mutant of DHHC9 (FLAG-DHHA9 Mut) and an untagged S protein and 48 h later, immunoprecipitated with FLAG antibodies. The respective empty vectors were used as controls. *HC, heavy chain of IgG. (**B**) Immunoprecipitation of Myc tagged Golga7 with S protein. HEK293T cells were transfected with Myc tagged Golga7 and an untagged S protein and 48 h later, immunoprecipitated with anti-Myc antibodies. (**C**). Co-localization of S protein with the cis-Golgi marker, GM130, or the endoplasmic reticulum marker, Calnexin. Vero-E6-ACE2 cells were infected with SARS-CoV-2 (MOI 0.01) and 48 h later, immunostained for the indicated proteins. (**D**) Similar experiment as in (**B**) showing co-localization between S protein and endogenous DHHC5 and DHHC9. (**E**) HEK293T cells were transfected with either untagged S protein with FLAG-DHHC9 or Myc-Golga7 and 48 h later, immunostained with anti-FLAG or anti-Myc antibodies together with anti-S antibodies, to show co-localization of transfected S protein with exogenous DHHC9 and Golga7. (**F**) Proximity ligation assay (PLA) to detect the physical proximity between the S protein and FLAG-DHHC9. Experiment was performed as in (**D**), and PLA was performed with the indicated antibodies followed by immunofluorescence (IFA) against either GM130 or Calnexin to visualize the subcellular localization of the detected PLA spots. (**G**) PLA to detect the physical proximity between the S protein and Myc-Golga7. Experiment same as in (**F**).

To determine whether DHHC9 interacts with the SARS-CoV-2 S protein, we performed a Co-IP experiment with FLAG tagged DHHC9 or its catalytic mutant, DHHA9 Mut, in HEK293T cells co-transfected with the S protein. DHHC9 and the S protein physically interact, and the interaction was not dependent on the palmitoyltransferase activity of DHHC9 (Figure 5A). In contrast, a similar Co-IP experiment with Myc tagged Golga7 failed to detect an interaction with the S protein (Figure 5B).

Next, to examine whether the S protein interacts with DHHC9 in infected cells, we performed immunofluorescence experiments in Vero-E6-ACE2 cells infected with SARS-CoV-2 for 48 h. Co-localization (yellow) with the endoplasmic reticulum marker, Calnexin and the cis-Golgi marker, GM130, indicates that the S protein localizes to both the ER and the Golgi apparatus (Figure 5C). Similar observations were made in HEK293T cells transfected with FLAG-DHHC9, Myc-Golga7 or the S protein, where all of these proteins were found to localize to the ER and the Golgi network (Figure S2A). Following this, we performed co-localization experiments between the S protein and DHHC5 and 9 in SARS-CoV-2 infected Caco-2 cells (Figure 5D). In agreement with previous observations [49,50], we observed DHHC5 to be localized predominantly on the cell surface, but in addition, some DHHC5 could be observed at other intracellular locations (Figure 5D). Because the S protein is primarily localized to the ER and the Golgi, we did not observe significant co-localization between S and DHHC5 (Figure 5D, upper panel). However, under similar conditions, DHHC9 extensively co-localized with S protein (Figure 5D, lower panel). To further confirm the S protein’s co-localization with DHHC9 and to exclude the possible non-specific staining due to the DHHC9 antibody, we performed similar co-localization experiments in HEK293T cells transfected with FLAG DHHC9 and Myc tagged Golga7. Significant co-localization was observed between DHHC9 and the S protein (Figure 5E, top panel). Myc-Golga7 also co-localized with the S protein, albeit to a lesser degree (Figure 5E, bottom panel).

The proximity ligation assay (PLA) provides a better method to assess direct interactions (<40 nm) between proteins in cells [51]. When PLA was performed, we observed robust interactions between the S protein and FLAG DHHC9 (Figure 5F). We also performed immunofluorescence against Calnexin and GM130 in the same experiment and found that the PLA signal between the S protein and DHHC9 localizes partially to the Golgi apparatus, but almost entirely to the ER network (Figure 5F, arrows). This suggests that palmitoylation of S occurs primarily in the ER and secondarily in the ER–Golgi intermediate compartment (ERGIC), as Calnexin is also found in the ERGIC [52,53]. In a similar experiment, where PLA was performed between the S protein and Golga7, we failed to detect a significant PLA signal, indicating again that S and Golga7 do not directly interact with each other (Figure 5G). The specificity of the PLA signal was confirmed by isotype IgG controlled PLA reaction which did not produce any signal (Figure S2B).

### 3.7. SARS-CoV-2 Infection and Syncytia Formation in Caco-2 Cells Requires DHHC9-Dependent Palmitoylation of S Protein

**Figure 6:**
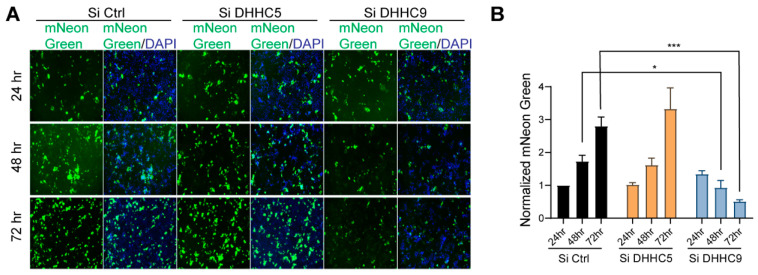
Effect of DHHC9 knockdown on SARS-CoV-2 infection in Caco-2 cells. (**A**) Caco-2 cells were knocked down for DHHC5 or DHHC9 and 48 h later, infected with icSARS-CoV-2-mNG (SARSCoV-2 stably encoding mNeonGreen; MOI 0.1). Then, 24, 48 and 72 h post-infection, the cells were fixed, nucleus stained with DAPI and visualized under a fluorescence microscope. (**B**) mNeonGreen signal from (**A**) was quantitated, normalized to DAPI and plotted to show the effect of the respective acyltransferase knocked down on icSARS-CoV-2-mNG infection. All data shown are averages of the results of at least three independent experiments (3 fields each) ± SD. * *p* < 0.05; *** *p* < 0.001 (*t*-test).

Having established a functional role for DHHC9 in palmitoylating the SARS-CoV-2 S protein using a pseudovirus system, we turned our attention to the infectious SARS-CoV-2 virus. SARS-CoV-2-mNG is a recombinant virus, derived from the 2019-nCoV/USA_WA1/2020 strain in which the mNeonGreen gene has been inserted into ORF7 of the viral genome [41]. The recombinant virus exhibits similar plaque morphology, viral RNA profile, and replication kinetics compared to the original clinical isolate [41]. This provides a more direct measure of the effect of S palmitoylation on the WT SARS-CoV-2 virus function. Caco-2 cells transfected with DHHC9 or DHHC5 siRNA were infected with SARS-CoV-2-mNG and 24, 48 and 72 h post-infection monitored for mNeonGreen expression. At 24 h post-infection, the mNeonGreen signal was not measurably reduced after knockdown of DHHC5 or DHHC9 (Figure 6A,B and Figure S3). However, at 48 and 72 h post-infection, the mNeonGreen signal in the DHHC9 knocked down cells reduced significantly. We reason that, as the cells were infected with SARS-CoV-2-mNG harvested from WT Vero-E6 cells, the S protein on these virus particles were efficiently palmitoylated and knockdown of the respective PATs in the recipient Caco-2 cells had no effect on the infection process and the expression of mNeonGreen at 24 h post-infection. However, with time (48 and 72 h), nascent virion particles increasingly harbored palmitoylation-deficient S protein which resulted in reduced mNeonGreen signal and lower levels of infection of neighboring cells, suggesting that DHHC9 plays a key role in the palmitoylation of the S protein during SARS-CoV-2 infection.

### 3.8. Novel Bis-Piperazine DHHC9 Inhibitors Inhibit SARS-CoV-2 S Palmitoylation, Fusogenicity and Infectivity

**Figure 7:**
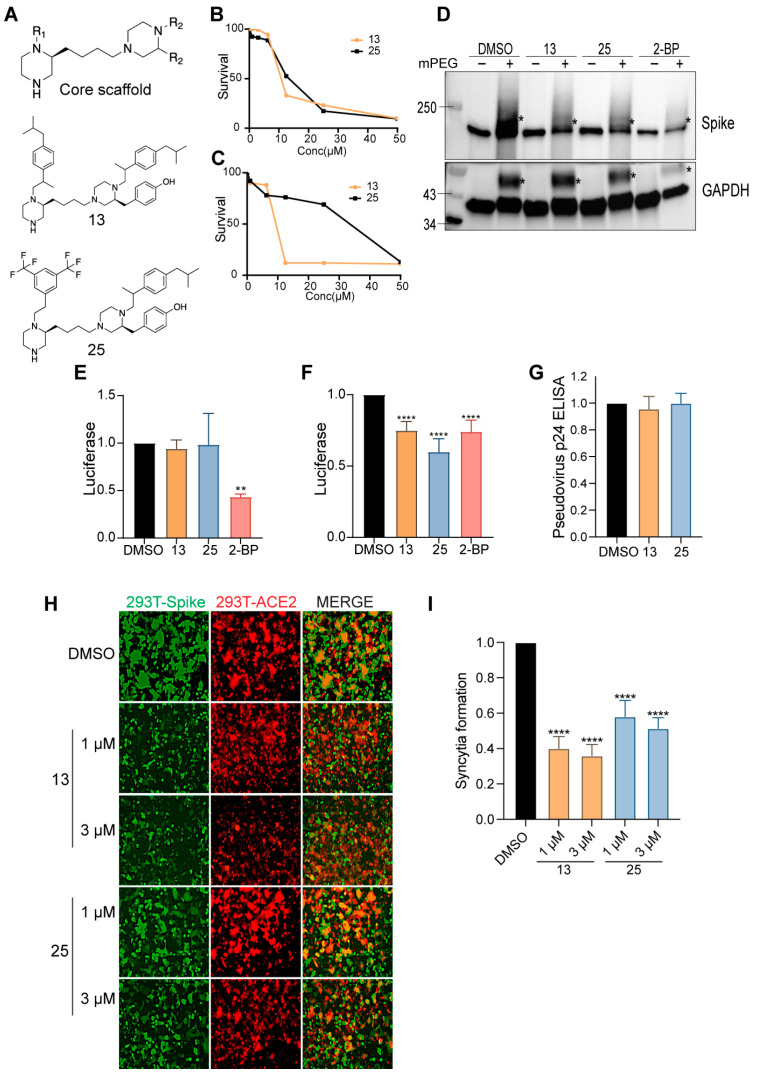
Inhibition of SARS-CoV-2 infectivity by DHHC9 inhibitors. (**A**) Chemical structures of compounds **13** and **25**. (**B**,**C**) XTT cell viability assay to access the toxicity of compounds **13** and **25** on HEK293T and Caco-2 cells, respectively. Survival is plotted relative to DMSO, vehicle only control. (**D**) APEGS assay to evaluate the palmitoylation of the WT S protein after treatment with compounds **13** and **25**. HEK293T cells were treated with the maximal non-toxic concentration of each compound (3 µM) or the broad spectrum, non-specific acyltransferase inhibitor 2-BP (10 µM) for 12 h before transfection with the S plasmid. APEGS assay was performed 48 h later. DHHC9-independent palmitoylation of GAPDH was included as a control. (**E**) HEK293T-ACE2 cells were pretreated with either compounds **13** (3 µM) or **25** (3 µM) or 2-BP (10 µM) for 12 h and then infected with luciferase reporter lentivirus pseudotyped with WT S protein. Luciferase activity was measured 48 h later. (**F**) HEK293T cells were pretreated with either compounds **13** (3 µM), **25** (3 µM) or 2-BP (10 µM) for 12 h and then transfected with plasmids required to produce luciferase reporter lentivirus pseudotyped with WT S protein. The pseudovirus collected was used to infect HEK293T-ACE2 cells and luciferase activity was measured after 48 h. (**G**). HEK293T cells were pretreated with compounds **13** (3 µM) or **25** (3 µM) or 2-BP (10 µM) and then transfected with plasmids required to produce lentivirus pseudotyped with WT S protein. The supernatants containing pseudotyped lentivirus particles were assayed 48 h later for pseudovirus egress by ELISA against the HIV p24 protein. (**H**) Dual fluorescent syncytia formation assay. HEK293T-EGFP cells were pretreated with the indicated concentrations of compounds **13** and **25** for 12 h and then transfected with WT S plasmid. The cells were detached and mixed 24 h later with HEK293T-ACE2 cells labeled with cell tracker red CMPTX dye. Syncytia formation was evaluated at 24 h by visualizing the yellow fluorescence formed by the fusion of the green and red cells. (**I**) Quantification of cell–cell fusion ability by measuring the pixel density of the observed syncytia in (H). All data shown are averages of the results of at least three independent experiments ± SD. * *p* < 0.05; ** *p* < 0.01; **** *p* < 0.0001 (*t*-test).

Having identified DHHC9 as a major SARS-CoV-2 S protein palmitoylating enzyme, we investigated whether inhibiting DHHC9 would inhibit SARS-CoV-2 infection. The availability of validated and specific PAT inhibitors is very limited [54]. 2-bromopalmitate (2-BP), the most widely used PAT inhibitor, promiscuously inhibits a wide range of enzyme utilizing active site cysteine residues [33,55,56,57]. Previously, using a scaffold ranking approach to screen for novel inhibitors of the yeast homolog of DHHC9, members of our group identified a number of bis-piperazine backbone-based compounds [35]. Two lead molecules from this study, compounds **13** and **25**, inhibited palmitoylation at low micromolar concentrations. Compound **13** has the lead functional group, 2-(3,5-bis-trifluoromethylphenyl)-ethyl, at positions R1 and R3, while compound **25** has 4-tert-butyl-cyclohexyl-methyl at the R1 position and 2-(3,5-bis-trifluoromethyl-phenyl)-ethyl at position R3 (Figure 7A). However, these compounds were not tested on mammalian cells before. We evaluated the palmitoylation inhibitory potential of compounds **13** and **25** against the SARS-CoV-2 S protein in Caco-2 cells.

The compounds were first tested for toxicity on HEK293T and Caco-2 cells and found to have no observable toxicity at concentrations below 3 µM (Figure 7B,C, respectively). We next examined whether compounds **13** and **25** inhibited the SARS-CoV-2 S protein palmitoylation using APEGS assay. Compared to the vehicle control, compounds **13** and **25** (3 µM) significantly reduced palmitoylation of the S protein in HEK293T cells (Figure 7D). In the same experiment, we found that GAPDH palmitoylation is reduced by the non-specific inhibitor, 2-BP, but not by compounds **13** and **25** (Figure 7D), consistent with the DHHC9 specific activity previously observed for these compounds [35].

We next examined the effect of these palmitoyltransferase inhibitors on the cellular entry and infectivity of SARS-CoV-2 S pseudotyped lentivirus on HEK293T-ACE2 cells. First, we treated the recipient HEK293T-ACE2 with the inhibitors and found that there was no reduction in luciferase signal upon treatment with compounds **13** and **25** (Figure 7E). However, 2-BP caused a significant reduction in pseudovirus infection in this experiment, implying that it interferes with the SARS-CoV-2 S protein-mediated lentivirus entry (Figure 7E). This also signifies that compounds **13** and **25** do not affect any step downstream of infection. In a second series of experiments, HEK293T cells were treated with compounds **13**, **25** or 2-BP and pseudovirus produced from these cells were tested for their ability to infect untreated HEK293T-ACE2 cells. In this case, we observed a significant reduction in the luciferase signal (Figure 7F), indicating that inhibition of S palmitoylation resulted in a pseudovirus with reduced ability to infect cells. Under similar conditions, 2-BP also reduced luciferase signal significantly. We also found that compounds **13** and **25** do not reduce the quantity of pseudovirus released from the producer cells, indicating that there is no measurable effect on lentivirus packaging and egress (Figure 7G). Furthermore, we observed that inhibition of S palmitoylation by compounds **13** and **25** reduces its fusogenicity in a syncytia formation assay. Treatment with compound **13** (1 µM and 3 µM) resulted in a 58% and 60% reduction in syncytia formation, respectively. Compound **25** (1 µM and 3 µM) similarly reduced syncytia formation by 45% and 50%, respectively (Figure 7H,I).

### 3.9. Compounds ***13*** and ***25*** Inhibit SARS-CoV-2 Infection in Cell Culture

**Figure 8:**
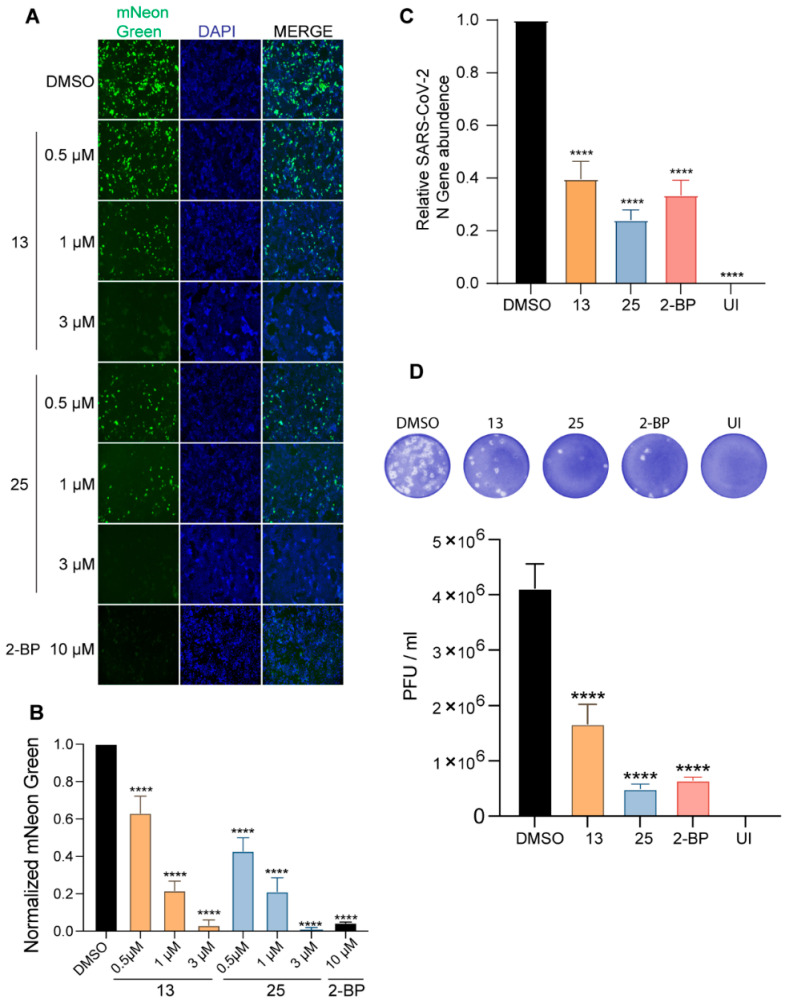
Compounds **13** and **25** inhibit SARS-CoV-2 infection. (**A**) Caco-2 cells were pretreated with the indicated concentrations of compounds **13** and **25** for 12 h and then infected with icSARS-CoV-2-mNG (MOI 0.1). Post-infection, the cells were continued to be incubated in the presence of the respective compound dilutions. The cells were fixed 72 h later and nucleus stained with DAPI and visualized under a fluorescence microscope; 10 µM 2-BP was also included in this experiment. (**B**) mNeonGreen signal from (**A**) was quantitated (6 fields from each experiment), normalized to DAPI and plotted to show the effect of the respective concentrations of the DHHC9 PAT inhibitors on icSARS-CoV-2-mNG infection. Data shown are averages of the results of at least three independent experiments ± SD (One-Way ANOVA). (**C**) Caco-2 cells were pretreated with compounds **13** (3 µM), **25** (3 µM) or 2-BP (10 µM) for 12 h and then infected with SARS-CoV-2 (MOI 0.1). Post-infection, the cells were continued to be incubated in the presence of the respective compound dilutions. The virus-containing supernatants were collected 72 h later and used to infect Vero-E6-ACE2 cells. The Vero-E6-ACE2 cells were collected 24 h after this infection, RNA extracted, and the SARS-CoV-2 N gene quantified using RT-qPCR. UI represents uninfected. (**D**) Same experiment as in C, except that the virus titers in the supernatant were estimated by plaque assay on VERO-ACE2-TMPRSS2 cells. All data shown are averages of the results of at least three independent experiments ± SD **** *p* < 0.0001 (*t*-test).

We tested the effect of these palmitoyltransferase inhibitors on SARS-CoV-2 infection using the SARS-CoV-2-mNG. Caco-2 cells pretreated with compounds **13**, **25**, or 2-BP and infected with SARS-CoV-2-mNG exhibited dose-dependent reduction in mNeonGreen signal after 72 h of infection (Figure 8A,B and Figure S4). The size of the syncytia was also reduced in the inhibitor treated cells compared to the control vehicle treated cells (Figure 8A). This is consistent with the conclusion that compounds **13** and **25** inhibit SARS-CoV-2 S protein palmitoylation, causing a reduction in the infection competent virus released and their ability to infect neighboring cells.

Next, virus-containing supernatants were collected 72 h after SARS-CoV-2 infection of Caco-2 cells pretreated with the inhibitors and used to infect Vero-E6-ACE2 cells. After 24 h, infection was quantitated either by measuring the abundance of the SARS-CoV-2 N gene by real-time RT-PCR (Figure 8C) or by plaque titration (Figure 8D). SARS-CoV-2 virus isolated from Caco-2 cells treated with compounds **13** and **25** resulted in a 60% and 76% reduction in viral infection as measured by qRT-PCR (Figure 8C). A comparable reduction of the SARS-CoV-2 titer was also observed through plaque reduction assay (Figure 8D), establishing the antiviral effects of compounds **13** and **25**.

## 4. Discussion

In this study, we demonstrate that the SARS-CoV-2 S protein is palmitoylated on a cluster of conserved cysteine residues on the cytosolic domains of the SARS-CoV-2 S protein. Mutating all 10 cysteine residues to serine (∆C) eliminates palmitoylation but mutating individual clusters of cysteines suggests that not all cysteine residues are palmitoylated equivalently. For example, mutating clusters C1 (C1235, C1236), C2 (C1240, C1241, C1243), and C4 (C1253, C1254) significantly reduces but does not eliminate S palmitoylation. In contrast, S palmitoylation is unaffected by mutating the C3 (C1248, C1249, C1250) cluster. Individual cysteine clusters are also functionally different. Mutating C1 and C2 clusters reduce infection of ACE-2 expressing cells to the same extent as ∆C, indicating that palmitoylation of the juxtamembrane cysteines are the most important for infection. Mutating the C3 cluster does not reduce palmitoylation or influence infection. Interestingly, mutating the C4 cluster reduces overall palmitoylation, but does not reduce infection, suggesting that palmitoylation of the C4 cysteines may have other roles in the viral life cycle.

Though palmitoylation has been established to play major roles in intracellular protein transport [18,58], the ∆C S protein still localized to the plasma membrane, suggesting that there are alternate trafficking routes independent of palmitoylation status. We have also shown that palmitoylation is not required for ACE2 receptor binding. This is expected, as ACE2 binding occurs via the RBD domain of the S protein, and the C-terminal palmitoylation sites do not structurally regulate this phenomenon. However, despite being capable of binding ACE2 and no decrease in membrane expression, viruses harboring non-palmitoylated S protein fail to infect host cells. One reason can be that membrane fusion depends on surface density of S proteins [59]. Palmitoylation-dependent clustering of the S protein in infected cell lipid rafts possibly can increase the S protein density on the membrane to support membrane fusion [24]. Alternatively, palmitoylation of S protein may play other, more direct roles in membrane fusion. Since the palmitoylation sites of the SARS-CoV S protein CRDs are in the vicinity of the membrane bilayer, it is likely that the palmitoylated cysteines will anchor the cytoplasmic tail to the membrane. The potential to palmitoylate up to 10 cysteine residues would create a stronger membrane anchor for the endodomain of the S protein. While it is possible that changes in the S protein endodomain via palmitoylation or mutation of the cysteine residues might destabilize the S protein, resulting in its proteasomal clearance, we do not think this is the case. First, we find that the wild-type S protein and its palmitoylation defective mutants are expressed at similar levels, and also traffic to the plasma membrane and bind to ACE2 at similar levels. Pre-fusion, the S protein typically exists in a metastable conformation. Once it interacts with the host ACE2 receptor, extensive structural rearrangement of the S protein occurs, allowing the virus to fuse with the host cell membrane [45,60]. This S2 domain directed fusion event requires a concerted cooperation between the different domains of the S protein trimers [61]. It is therefore possible that conformational changes mediated by palmitoylation in the cytoplasmic endodomain of the S protein impact the ability of the extracellular ectodomain to adopt the proper conformation required for efficient cell fusion, implying a cooperation between the ecto and endodomains. In addition, extensive palmitoylation of the CRD could also be required to create a stable anchor during the membrane fusion process. One or more of these mechanisms likely results in the failure of non-palmitoylated S proteins to carry out efficient membrane fusion.

S-acylation of viral proteins requires the host cell palmitoylation machinery that, depending on the cell type, consists of up to 23 individual DHHC PAT genes. Identification of the DHHC enzyme or enzymes that palmitoylate the SARS-CoV-2 S protein has begun to emerge from several lines of investigation. First, a comprehensive interactome study uncovered an interaction between the SARS-CoV-2 S protein and Golga7, an auxiliary protein for DHHC5, DHHC9, and possible additional DHHC proteins [31]. Subsequently, knockdown and overexpression experiments showed that DHHC8, 9, and 20 are implicated in the palmitoylation of the SARS-CoV-2 S protein [8]. Other concurrent studies identified even more PAT enzymes as being involved in this palmitoylation—DHHCs 2, 3, 4, 5, 8, 9, 11, 14, 16, 19, and 20 by Li et al. [9] and DHHCs 2, 3, 6, 11, 20, 21, and 24 by Puthenveetil et al. [5]. This suggests that although it is certain that multiple PATs are involved in S protein palmitoylation, the specific role of each palmitoyltransferase requires additional work. Interestingly, DHHC9 and 20 are expressed in all animal reservoirs of Coronaviruses [62] and are therefore most likely to play major roles in the regulation of the S protein palmitoylation. Intriguingly, these two PATs were also among the ones that were upregulated when we knocked down DHHC5. Though the reason for this is unclear, it may suggest cross-talk between these PATs. In this study, we focused on DHHC9 and used several approaches to show that DHHC9 plays a major role in palmitoylating the SARS-CoV-2 S protein. Co-localization and proximity ligation studies confirm that the S protein and DHHC9 interacts in the ER, Golgi and the ERGIC. However, as co-localization or physical interaction of a particular DHHC enzyme with a palmitoylated protein does not always confirm its involvement in the palmitoylation process [63], we conducted DHHC9 knockdown experiments. Knockdown of DHHC9 resulted in a decrease in S protein palmitoylation, pseudovirus fusion, syncytia formation, and a 55% and 80% reduction of SARS-CoV-2 infection at 48 h and 72 h post-inoculation, respectively, establishing its role in S protein palmitoylation. However, we cannot rule out that palmitoylation of the S protein by other DHHC PATs also occurs during the viral life cycle.

Our work and that of several other groups have led to the suggestion that inhibitors of palmitoylation may be developed into a new class of antivirals [29,30,31]. To do so will require identification of high affinity, specific inhibitors of DHHC PATs. This has proved to be difficult. Commonly used inhibitors such as 2-bromopalmitte exhibit little specificity, inhibiting a wide range of thiol-containing enzymes [33,55]. We previously developed a high-throughput palmitoylation assay and used it to screen a chemical library consisting of 68 unique scaffolds and 30 million unique structures for inhibition of the yeast ortholog of DHHC9 [35]. Two compounds based on a bis-piperazine backbone (compounds **13** and **25**) were selected to be tested for inhibition of SARS-CoV-2 S protein palmitoylation and viral infection. Using both the pseudovirus model and wild-type SARS-CoV-2 virus, we show that compounds **13** and **25** decreased S protein palmitoylation, syncytia formation, and viral infection. Interestingly, DHHC9 has been reported to be highly expressed in the lungs, brain, kidney, skeletal muscle, and liver, and to a lesser extent in placenta, heart, colon, and small intestine [47], many of which are known to be tissues permissive to SARS-CoV-2 infection. Moreover, because the S proteins of many zoonotic viruses are palmitoylated [62], DHHC inhibitors may have broad antiviral applications. Further work is needed to establish the potential of DHHC PAT inhibitors to be developed into therapeutic drugs.

## Figures and Tables

**Table 1:** Mutagenic primers used.

Primer	Sequence	Source
C1_F	TATAATGCTGAGCAGCATGACAAGCTG	This Study
C1_R	GTCACCATGACTATAGCG	This Study
C2_F	AGCAGTCTCAAAGGCTGTTGCTCTTG	This Study
C2_R	GCTGCTGCTTGTCATGCAGCACAG	This Study
C3_F	TCTAGCGGCTCTTGCTGCAAATTC	This Study
C3_R	GCTACTGCCTTTGAGACAGCTGCA	This Study
C4_F	TTGCGGCTCTAGCAGCAAATTCGATG	This Study
C4_R	GAGCAACAGCCTTTGAGAC	This Study
ΔC_F	AAAGGCAGTAGCTCTAGCGGCTCTAGCAGCAAATTCGATGAGGACGATTC	This Study
ΔC_R	GAGACTGCTGCTGCTGCTTGTCATGCTGCTCAGCATTATAGTCACCATG	This Study

**Table 2:** Primary antibodies used.

Antibody	Source	Catalog Number
Spike S2	GeneTex	GTX632604
Spike S1	Sino Biological	40591-MM42
ACE2	Novus	NBP2-67692
GAPDH	Santa Cruz	sc-47724
Calnexin	Novus	NB300-518
Calnexin	Cell Signaling	2679P
FLAG	Sigma-Aldrich	F1804
FLAG	Sigma-Aldrich	F7425
Myc	Cell Signaling	71D10
Myc	Cell Signaling	9B11

**Table 3:** Real-time primers used.

Primer	Sequence1	Sequence2	Source
DHHC1	CAAGCCCTCCAACAAGACG	CCAAAGCCGATCACAGCAAAG	[39]
DHHC2	AACACTGGCGAACAAGTTGTG	AGATGGGAAGATCCTTGGCTG	[39]
DHHC3	CCACTTCCGAAACATTGAGCG	CCACAGCCGTCACGGATAAA	[39]
DHHC4	CCTGACTTGTGGAACCAATCC	GCACCTCACGTTCTTTGGAAAC	[39]
DHHC5	CACCTGCCGCTTTTACCGT	CGGCGACCAATACAGTTATTCAC	[39]
DHHC6	AGTCTGCCAAGCATACAAGGC	CCAGTGGTGCTAAAAGGAGAAAC	[39]
DHHC7	CTGACCGGGTCTGGTTCATC	CATGACGAAAGTCACCACGAA	[39]
DHHC8	GTATCCAGGTCCGCATGAAGT	AGCGTGGTTCAGCACGTAG	[39]
DHHC9	CCCAGGCAGGAACACCTTTT	CCGAGGAATCACTCCAGGG	[39]
DHHC11	GGTGCAGACCCTGATAGTCG	GCACGTATGGATCTTTCCTCAC	[39]
DHHC12	GTGCTGACCTGGGGAATCAC	CTGCACATTCACGTAGCCA	[39]
DHHC13	ACCCCACTCTTATTGATGGAGA	TGTCTGCCCATTTACATCTGTC	[39]
DHHC14	TGTGATAACTGCGTAGAACGGT	CGTGGGTGATAACGAATGCAA	[39]
DHHC15	GGTGCCAGTGCTCGTTATTGT	AAGACGTAGGCATAGTAGGACC	[39]
DHHC16	ACTCCGGGGTCTAGTACAGC	CCAGCGGATCACGTTGTCT	[39]
DHHC17	GGCCCGGATGAGTACGATAC	TCCAAGAGGTTCACCATATCCA	[39]
DHHC18	TGACGGCCTTCATCTTCGC	CTGGACCACGAGCCTTTGAT	[39]
DHHC19	TTGCTGCCTTCAATGTGGTG	CGGAGCCTTGATGTAAGATGC	[39]
DHHC20	CGCACCCACGTTTTCATACG	TCTGGCATACTCATTCTGGTTTG	[39]
DHHC21	TGTTGTTGACCCACATGGTTG	GAGGCCCTCACTAAGGCAA	[39]
DHHC22	GAGGCACGACCATCACTGTTT	ACAGCGGAGATGTAGGCCA	[39]
DHHC23	TCTGGATGAAGGGTGTGATCG	GCTCCCCTAAGCCAAGGAA	[39]
DHHC24	CTGGCACAGTTTGCCTTGG	CAGGGACCCAGGTCATAGGAG	[39]
SARS-CoV-2 N	CACATTGGCACCCGCAATC	GAGGAACGAGAAGAGGCTTG	[40]

## Supplementary Materials

The following supporting information can be downloaded at: https://www.mdpi.com/article/10.3390/v14030531/s1, Figure S1: Supplemental image to Figure 2B. Figure S2: Co-localization of FLAG-DHHC9, Myc-Golga7 and S protein with the cis-Golgi marker, GM130 or the endoplasmic reticulum marker, Calnexin, and control PLA with isotype IgGs showing absence of non-specific PLA signal. Figure S3: Supplemental image to Figure 6A. Figure S4: Supplemental image to Figure 8A.

## References

[1] Walls A.C., Park Y.J., Tortorici M.A., Wall A., McGuire A.T., Veesler D. (2020). Structure, Function, and Antigenicity of the SARS-CoV-2 Spike Glycoprotein. Cell.

[2] Nguyen H.T., Zhang S., Wang Q., Anang S., Wang J., Ding H., Kappes J.C., Sodroski J. (2020). Spike glycoprotein and host cell determinants of SARS-CoV-2 entry and cytopathic effects. J. Virol..

[3] Qing E., Kicmal T., Kumar B., Hawkins G.M., Timm E., Perlman S., Gallagher T. (2021). Dynamics of SARS-CoV-2 Spike Proteins in Cell Entry: Control Elements in the Amino-Terminal Domains. mBio.

[4] Zhou T., Tsybovsky Y., Gorman J., Rapp M., Cerutti G., Chuang G.Y., Katsamba P.S., Sampson J.M., Schon A., Bimela J. (2020). Cryo-EM Structures of SARS-CoV-2 Spike without and with ACE2 Reveal a pH-Dependent Switch to Mediate Endosomal Positioning of Receptor-Binding Domains. Cell Host Microbe.

[5] Puthenveetil R., Lun C.M., Murphy R.E., Healy L.B., Vilmen G., Christenson E.T., Freed E.O., Banerjee A. (2021). S-acylation of SARS-CoV-2 Spike Protein: Mechanistic Dissection, In Vitro Reconstitution and Role in Viral Infectivity. J. Biol. Chem..

[6] Wu Z., Zhang Z., Wang X., Zhang J., Ren C., Li Y., Gao L., Liang X., Wang P., Ma C. (2021). Palmitoylation of SARS-CoV-2 S protein is essential for viral infectivity. Signal. Transduct Target. Ther..

[7] Zeng X.T., Yu X.T., Cheng W. (2021). The interactions of ZDHHC5/GOLGA7 with SARS-CoV-2 spike (S) protein and their effects on S protein’s subcellular localization, palmitoylation and pseudovirus entry. Virol. J..

[8] Mesquita F.S., Abrami L., Sergeeva O., Turelli P., Qing E., Kunz B., Raclot C., Paz Montoya J., Abriata L.A., Gallagher T. (2021). S-acylation controls SARS-CoV-2 membrane lipid organization and enhances infectivity. Dev. Cell.

[9] Li D., Liu Y., Lu Y., Gao S., Zhang L. (2022). Palmitoylation of SARS-CoV-2 S protein is critical for S-mediated syncytia formation and virus entry. J. Med. Virol..

[10] Iwanaga T., Tsutsumi R., Noritake J., Fukata Y., Fukata M. (2009). Dynamic protein palmitoylation in cellular signaling. Prog. Lipid. Res..

[11] Mitchell D.A., Vasudevan A., Linder M.E., Deschenes R.J. (2006). Protein palmitoylation by a family of DHHC protein S-acyltransferases. J. Lipid Res..

[12] Gottlieb C.D., Linder M.E. (2017). Structure and function of DHHC protein S-acyltransferases. Biochem. Soc. Trans..

[13] Schmidt M.F., Bracha M., Schlesinger M.J. (1979). Evidence for covalent attachment of fatty acids to Sindbis virus glycoproteins. Proc. Natl Acad. Sci. USA.

[14] Schmidt M.F., Schlesinger M.J. (1979). Fatty acid binding to vesicular stomatitis virus glycoprotein: A new type of post-translational modification of the viral glycoprotein. Cell.

[15] Lobo S., Greentree W.K., Linder M.E., Deschenes R.J. (2002). Identification of a Ras palmitoyltransferase in Saccharomyces cerevisiae. J. Biol. Chem..

[16] Roth A.F., Feng Y., Chen L., Davis N.G. (2002). The yeast DHHC cysteine-rich domain protein Akr1p is a palmitoyl transferase. J. Cell. Biol..

[17] Blanc M., David F.P.A., van der Goot F.G. (2019). SwissPalm 2: Protein S-Palmitoylation Database. Methods Mol. Biol..

[18] Linder M.E., Deschenes R.J. (2007). Palmitoylation: Policing protein stability and traffic. Nat. Rev. Mol. Cell Biol..

[19] Jiang H., Zhang X., Chen X., Aramsangtienchai P., Tong Z., Lin H. (2018). Protein Lipidation: Occurrence, Mechanisms, Biological Functions, and Enabling Technologies. Chem. Rev..

[20] Veit M. (2012). Palmitoylation of virus proteins. Biol. Cell.

[21] Thorp E.B., Boscarino J.A., Logan H.L., Goletz J.T., Gallagher T.M. (2006). Palmitoylations on murine coronavirus spike proteins are essential for virion assembly and infectivity. J. Virol..

[22] Yang J., Lv J., Wang Y., Gao S., Yao Q., Qu D., Ye R. (2012). Replication of murine coronavirus requires multiple cysteines in the endodomain of spike protein. Virology.

[23] Petit C.M., Chouljenko V.N., Iyer A., Colgrove R., Farzan M., Knipe D.M., Kousoulas K.G. (2007). Palmitoylation of the cysteine-rich endodomain of the SARS-coronavirus spike glycoprotein is important for spike-mediated cell fusion. Virology.

[24] McBride C.E., Machamer C.E. (2010). Palmitoylation of SARS-CoV S protein is necessary for partitioning into detergent-resistant membranes and cell-cell fusion but not interaction with M protein. Virology.

[25] Levental I., Levental K.R., Heberle F.A. (2020). Lipid rafts: Controversies resolved, mysteries remain. Trends Cell Biol..

[26] Buchrieser J., Dufloo J., Hubert M., Monel B., Planas D., Rajah M.M., Planchais C., Porrot F., Guivel-Benhassine F., Van der Werf S. (2020). Syncytia formation by SARS-CoV-2-infected cells. EMBO J..

[27] Cattin-Ortola J., Welch L.G., Maslen S.L., Papa G., James L.C., Munro S. (2021). Sequences in the cytoplasmic tail of SARS-CoV-2 Spike facilitate expression at the cell surface and syncytia formation. Nat. Commun..

[28] Lin L., Li Q., Wang Y., Shi Y. (2021). Syncytia formation during SARS-CoV-2 lung infection: A disastrous unity to eliminate lymphocytes. Cell Death Differ..

[29] Gadalla M.R., Veit M. (2020). Toward the identification of ZDHHC enzymes required for palmitoylation of viral protein as potential drug targets. Expert Opin. Drug Discov..

[30] Santos-Beneit F., Raskevicius V., Skeberdis V.A., Bordel S. (2021). A metabolic modeling approach reveals promising therapeutic targets and antiviral drugs to combat COVID-19. Sci. Rep..

[31] Gordon D.E., Jang G.M., Bouhaddou M., Xu J., Obernier K., White K.M., O’Meara M.J., Rezelj V.V., Guo J.Z., Swaney D.L. (2020). A SARS-CoV-2 protein interaction map reveals targets for drug repurposing. Nature.

[32] Lee M., Sugiyama M., Mekhail K., Latreille E., Khosraviani N., Wei K., Lee W.L., Antonescu C., Fairn G.D. (2020). Fatty Acid Synthase inhibition prevents palmitoylation of SARS-CoV2 Spike Protein and improves survival of mice infected with murine hepatitis virus. bioRxiv.

[33] Davda D., El Azzouny M.A., Tom C.T., Hernandez J.L., Majmudar J.D., Kennedy R.T., Martin B.R. (2013). Profiling targets of the irreversible palmitoylation inhibitor 2-bromopalmitate. ACS Chem. Biol..

[34] Chavda B., Arnott J.A., Planey S.L. (2014). Targeting protein palmitoylation: Selective inhibitors and implications in disease. Expert Opin. Drug Discov..

[35] Hamel L.D., Lenhart B.J., Mitchell D.A., Santos R.G., Giulianotti M.A., Deschenes R.J. (2016). Identification of Protein Palmitoylation Inhibitors from a Scaffold Ranking Library. Comb. Chem. High Throughput Screen.

[36] Dull T., Zufferey R., Kelly M., Mandel R.J., Nguyen M., Trono D., Naldini L. (1998). A third-generation lentivirus vector with a conditional packaging system. J. Virol..

[37] Crawford K.H.D., Eguia R., Dingens A.S., Loes A.N., Malone K.D., Wolf C.R., Chu H.Y., Tortorici M.A., Veesler D., Murphy M. (2020). Protocol and Reagents for Pseudotyping Lentiviral Particles with SARS-CoV-2 Spike Protein for Neutralization Assays. Viruses.

[38] Percher A., Thinon E., Hang H. (2017). Mass-Tag Labeling Using Acyl-PEG Exchange for the Determination of Endogenous Protein S-Fatty Acylation. Curr Protoc. Protein Sci..

[39] McClafferty H., Shipston M.J. (2019). siRNA Knockdown of Mammalian zDHHCs and Validation of mRNA Expression by RT-qPCR. Methods Mol. Biol..

[40] Corman V.M., Landt O., Kaiser M., Molenkamp R., Meijer A., Chu D.K., Bleicker T., Brunink S., Schneider J., Schmidt M.L. (2020). Detection of 2019 novel coronavirus (2019-nCoV) by real-time RT-PCR. Euro Surveill..

[41] Xie X., Muruato A., Lokugamage K.G., Narayanan K., Zhang X., Zou J., Liu J., Schindewolf C., Bopp N.E., Aguilar P.V. (2020). An Infectious cDNA Clone of SARS-CoV-2. Cell Host Microbe.

[42] Rodenburg R.N.P., Snijder J., van de Waterbeemd M., Schouten A., Granneman J., Heck A.J.R., Gros P. (2017). Stochastic palmitoylation of accessible cysteines in membrane proteins revealed by native mass spectrometry. Nat. Commun..

[43] Ren J., Wen L., Gao X., Jin C., Xue Y., Yao X. (2008). CSS-Palm 2.0: An updated software for palmitoylation sites prediction. Protein Eng. Des. Sel..

[44] Yang J., Gibson B., Snider J., Jenkins C.M., Han X., Gross R.W. (2005). Submicromolar concentrations of palmitoyl-CoA specifically thioesterify cysteine 244 in glyceraldehyde-3-phosphate dehydrogenase inhibiting enzyme activity: A novel mechanism potentially underlying fatty acid induced insulin resistance. Biochemistry.

[45] Huang Y., Yang C., Xu X.F., Xu W., Liu S.W. (2020). Structural and functional properties of SARS-CoV-2 spike protein: Potential antivirus drug development for COVID-19. Acta Pharmacol. Sin..

[46] Scudellari M. (2021). How the coronavirus infects cells—And why Delta is so dangerous. Nature.

[47] Swarthout J.T., Lobo S., Farh L., Croke M.R., Greentree W.K., Deschenes R.J., Linder M.E. (2005). DHHC9 and GCP16 constitute a human protein fatty acyltransferase with specificity for H- and N-Ras. J. Biol. Chem..

[48] Ohta E., Misumi Y., Sohda M., Fujiwara T., Yano A., Ikehara Y. (2003). Identification and characterization of GCP16, a novel acylated Golgi protein that interacts with GCP170. J. Biol. Chem..

[49] Chen J.J., Marsden A.N., Scott C.A., Akimzhanov A.M., Boehning D. (2020). DHHC5 Mediates beta-Adrenergic Signaling in Cardiomyocytes by Targeting Galpha Proteins. Biophys. J..

[50] Woodley K.T., Collins M.O. (2019). S-acylated Golga7b stabilises DHHC5 at the plasma membrane to regulate cell adhesion. EMBO Rep..

[51] Fredriksson S., Gullberg M., Jarvius J., Olsson C., Pietras K., Gustafsdottir S.M., Ostman A., Landegren U. (2002). Protein detection using proximity-dependent DNA ligation assays. Nat. Biotechnol..

[52] Tsukamoto H., Tousson A., Circolo A., Marchase R.B., Volanakis J.E. (2002). Calnexin is associated with and induced by overexpressed human complement protein C2. Anat. Rec..

[53] Myhill N., Lynes E.M., Nanji J.A., Blagoveshchenskaya A.D., Fei H., Carmine Simmen K., Cooper T.J., Thomas G., Simmen T. (2008). The subcellular distribution of calnexin is mediated by PACS-2. Mol. Biol. Cell.

[54] Draper J.M., Smith C.D. (2009). Palmitoyl acyltransferase assays and inhibitors (Review). Mol. Membr. Biol..

[55] Coleman R.A., Rao P., Fogelsong R.J., Bardes E.S. (1992). 2-Bromopalmitoyl-CoA and 2-bromopalmitate: Promiscuous inhibitors of membrane-bound enzymes. Biochim. Biophys. Acta.

[56] Chase J.F., Tubbs P.K. (1972). Specific inhibition of mitochondrial fatty acid oxidation by 2-bromopalmitate and its coenzyme A and carnitine esters. Biochem. J..

[57] Pedro M.P., Vilcaes A.A., Tomatis V.M., Oliveira R.G., Gomez G.A., Daniotti J.L. (2013). 2-Bromopalmitate reduces protein deacylation by inhibition of acyl-protein thioesterase enzymatic activities. PLoS ONE.

[58] Greaves J., Chamberlain L.H. (2007). Palmitoylation-dependent protein sorting. J. Cell Biol..

[59] Bentz J., Mittal A. (2003). Architecture of the influenza hemagglutinin membrane fusion site. Biochim. Biophys. Acta.

[60] Wrapp D., Wang N., Corbett K.S., Goldsmith J.A., Hsieh C.L., Abiona O., Graham B.S., McLellan J.S. (2020). Cryo-EM structure of the 2019-nCoV spike in the prefusion conformation. Science.

[61] Shulla A., Gallagher T. (2009). Role of spike protein endodomains in regulating coronavirus entry. J. Biol. Chem..

[62] Abdulrahman D.A., Meng X., Veit M. (2021). S-Acylation of Proteins of Coronavirus and Influenza Virus: Conservation of Acylation Sites in Animal Viruses and DHHC Acyltransferases in Their Animal Reservoirs. Pathogens.

[63] Zaballa M.E., van der Goot F.G. (2018). The molecular era of protein S-acylation: Spotlight on structure, mechanisms, and dynamics. Crit Rev. Biochem. Mol. Biol..

