# Supplementary material for: Identification of SARS-CoV-2 Spike Palmitoylation Inhibitors That Results in Release of Attenuated Virus with Reduced Infectivity
Source: Viruses. 2022 Mar 4;14(3):531. doi: 10.3390/v14030531 (PMC8950683; doi:10.3390/v14030531)
Supplement: Supplementary file 1 [file viruses-14-00531-s001.zip › viruses-1604911-supplementary.pdf]

Supplementary Materials

Identification of SARS-CoV-2 spike palmitoylation inhibitors that results in release of attenuated virus with reduced infectivity

Ahmed A. Ramadan[1], Karthick Mayilsamy[1,3], Andrew R. McGill[1,2,3], Anandita Ghosh[1], Marc A. Giulianotti[4], Haley M. Donow[4], Shyam S. Mohapatra[1,2,3], Subhra Mohapatra[1,3], Bala Chandran[1], Robert J. Deschenes*[1], Arunava Roy*[1]

[1]Department of Molecular Medicine, University of South Florida, Tampa, FL 33612.

[2]Department of Internal Medicine, University of South Florida, Tampa, FL 33612.

[3]Department of Veterans Affairs, James A Haley Veterans Hospital, Tampa, FL, 33612.

[4]Center for Translational Science, Florida International University, Port St. Lucie, FL 34987

Figure S1

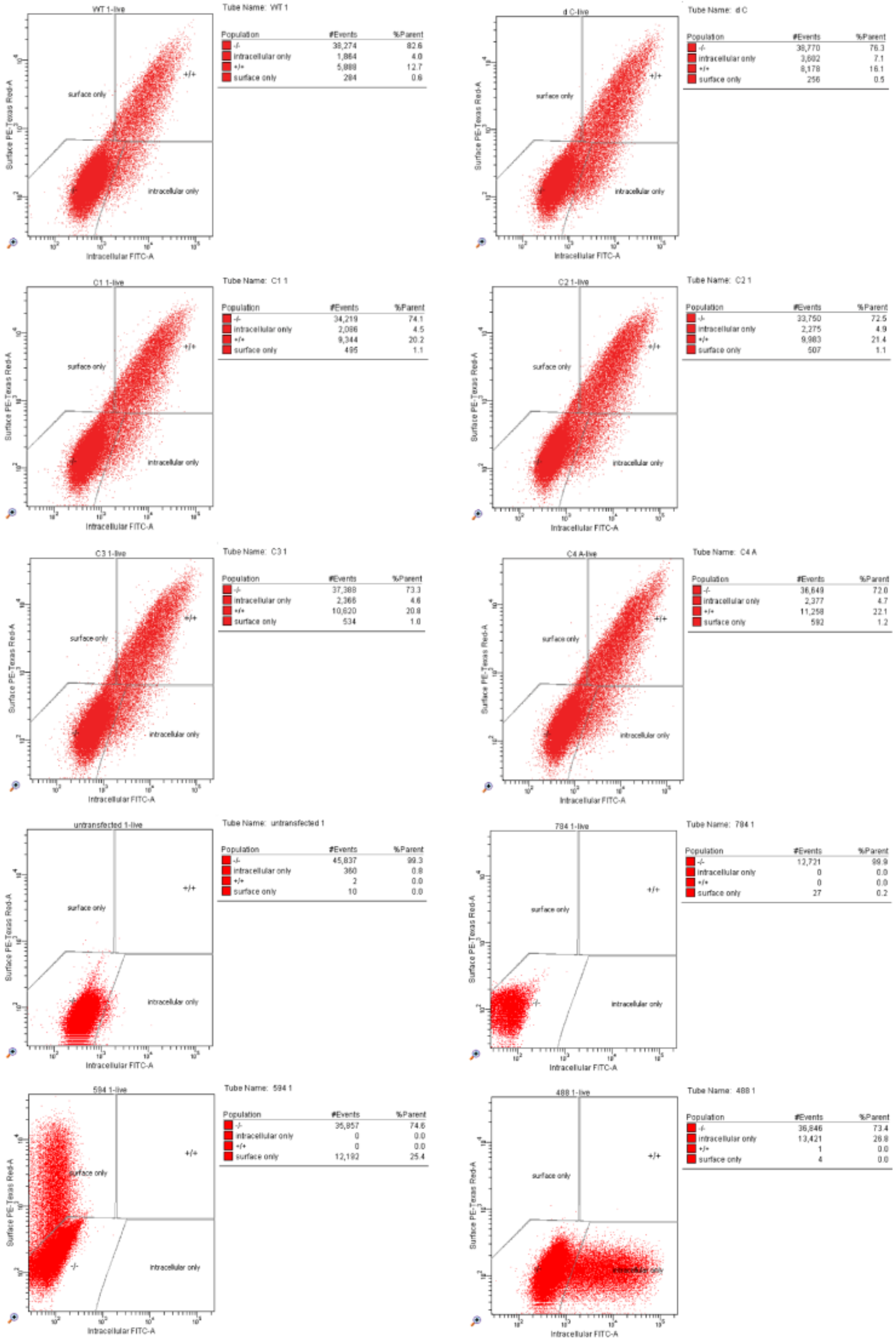

Supplemental image to Fig. 2B. Images of the Flow gates for the Spike-WT and its mutants in addition to the corresponding controls and fluorophore compensations.

Figure S2

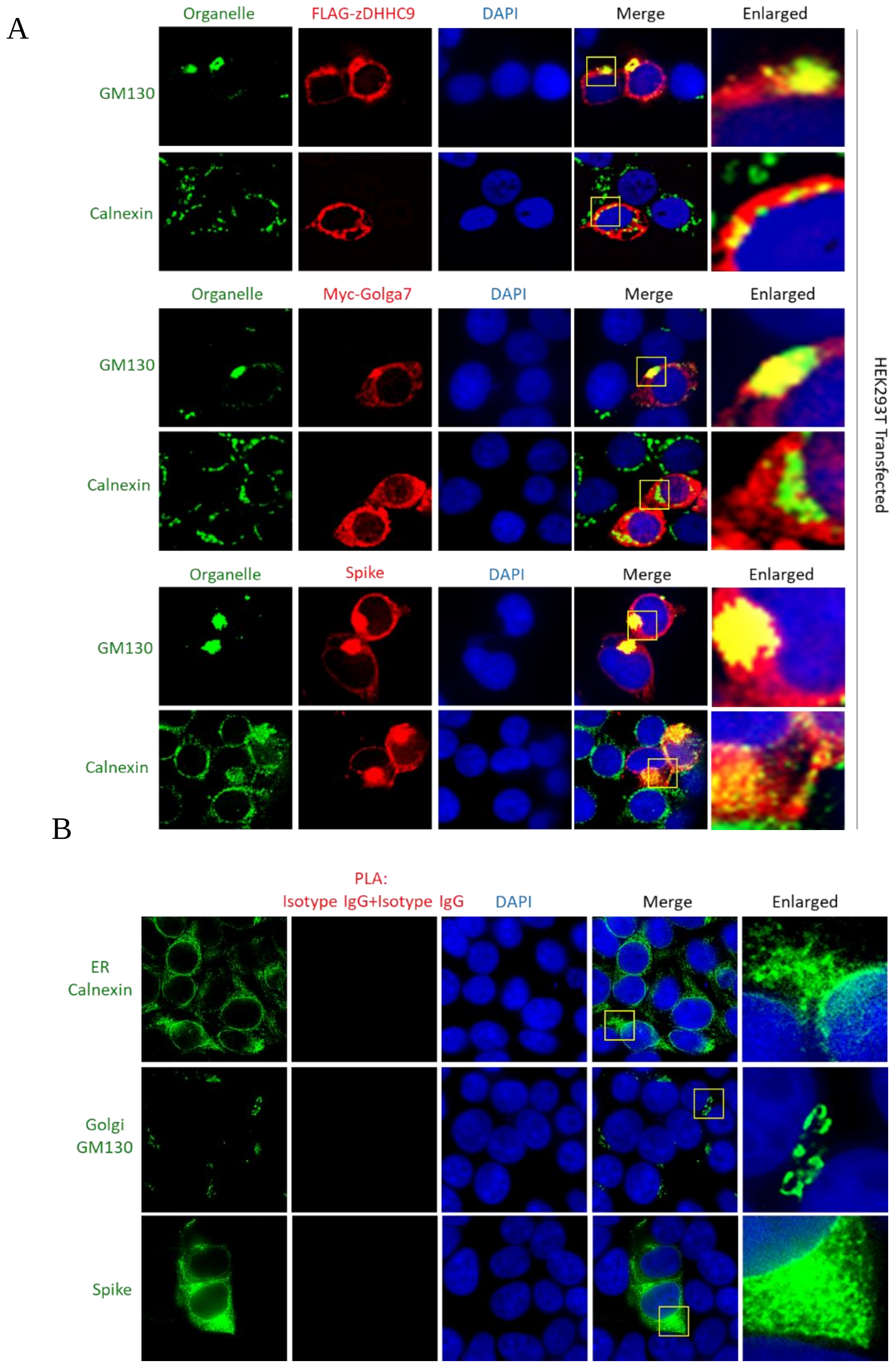

A. Co-localization of FLAG-DHHC9, Myc-Golga7 and S protein with the cis-Golgi marker, GM130 or the endoplasmic reticulum marker, Calnexin. HEK293T2 cells were transfected with the indicated plasmids and 72 h later, immunostained for the indicated proteins. **B.** Control PLA with isotype IgGs showing absence of non-specific PLA signal.

Figure S3

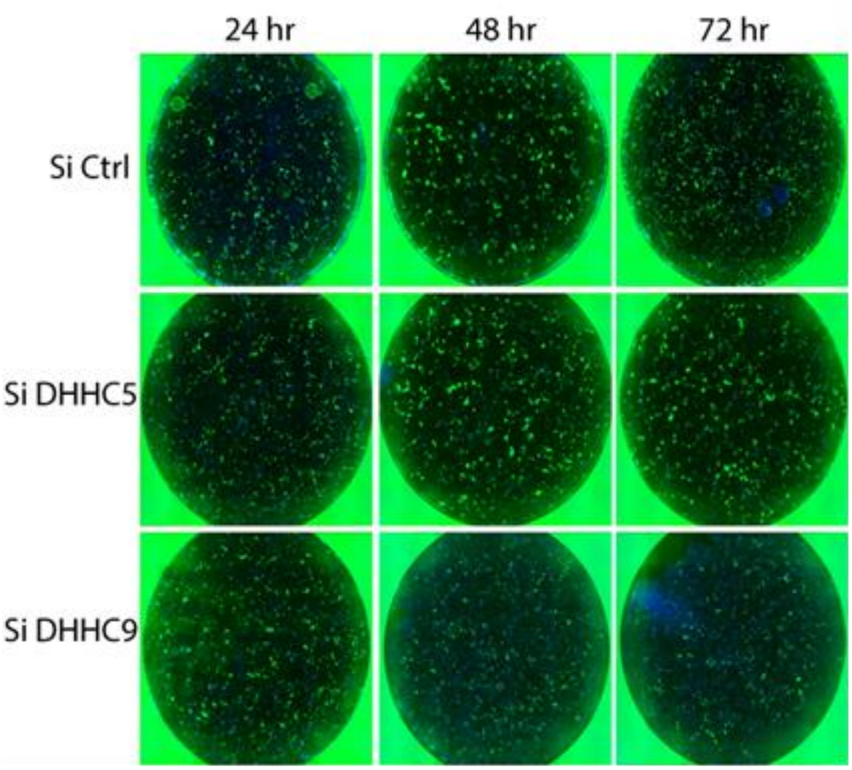

Supplemental image to Fig. 6A. Images of the entire well of the 96 well plate is provided to forego any bias in the region of interest (ROI) photographed.

Figure S4

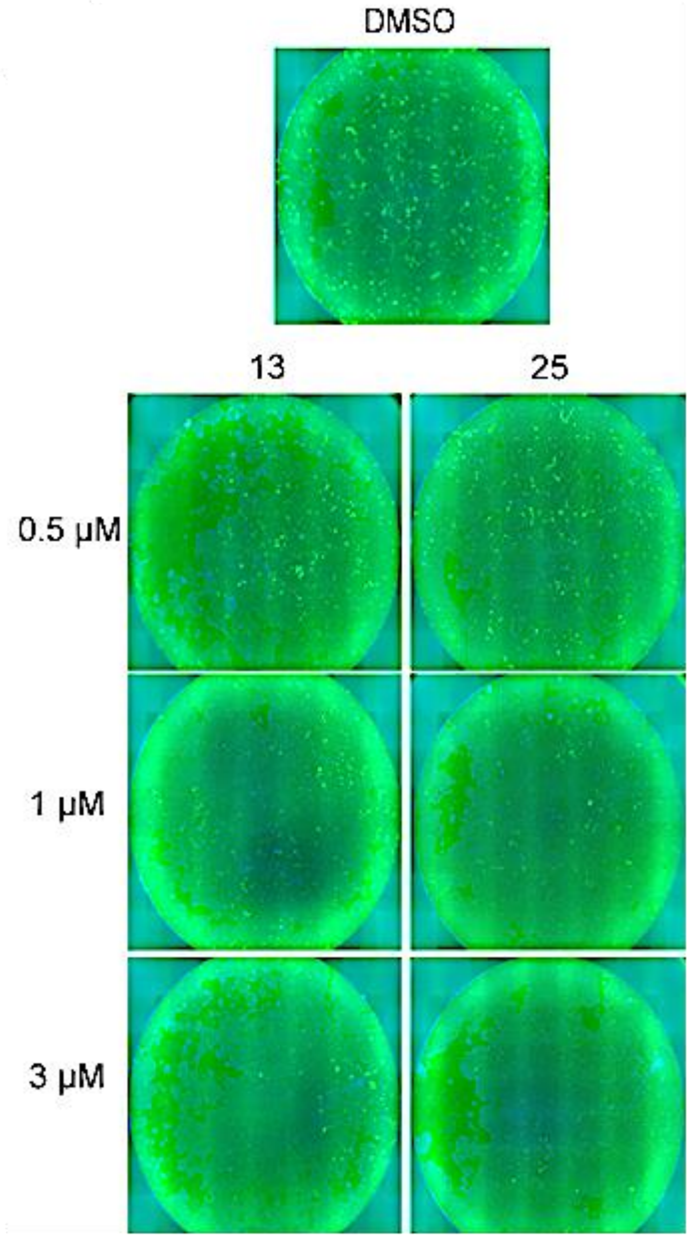

Supplemental image to Fig. 8A. Images of the entire well of the 96 well plate is provided to forego any bias in the ROI photographed.
